# Fungal microbiomes associated with Lycopodiaceae during ecological succession

**DOI:** 10.1111/1758-2229.13130

**Published:** 2022-10-10

**Authors:** Benoît Perez‐Lamarque, Liam Laurent‐Webb, Amélia Bourceret, Louis Maillet, Francis Bik, Denis Cartier, François Labolle, Pascal Holveck, Didier Epp, Marc‐André Selosse

**Affiliations:** ^1^ Institut de Systématique, Évolution, Biodiversité (ISYEB), Muséum national d'histoire naturelle, CNRS, Sorbonne Université, EPHE, UA, CP39 Paris France; ^2^ Institut de biologie de l'École normale supérieure (IBENS), École normale supérieure, CNRS, INSERM, Université PSL Paris France; ^3^ Société Botanique d'Alsace Sélestat France; ^4^ Pôle Lorrain du Futur Conservatoire Botanique National Nord‐Est, Jardin botanique Jean‐Marie Pelt Villers‐lès‐Nancy France; ^5^ Université de Strasbourg, Faculté des Sciences de la Vie, Institut de Botanique Strasbourg France; ^6^ Réseau National Habitats‐Flore, Office National des Forêts (ONF) Paris France; ^7^ Office National des Forêts (ONF), Service environnement et planification forestière Schirmeck France; ^8^ Department of Plant Taxonomy and Nature Conservation University of Gdansk Gdansk Poland; ^9^ Institut universitaire de France (IUF) Paris France

## Abstract

Lycopodiaceae species form an early‐diverging plant family, characterized by achlorophyllous and subterranean gametophytes that rely on mycorrhizal fungi for their nutrition. Lycopodiaceae often emerge after a disturbance, like in the Hochfeld reserve (Alsace, France) where seven lycopod species appeared on new ski trails following a forest cut. Here, to better understand their ecological dynamic, we conducted a germination experiment of lycopod spores following an anthropogenic disturbance and examined their associated fungi. Only 12% of the samples germinated, and all gametophytes were abundantly colonized by a specific clade of Densosporaceae (Endogonales, Mucoromycotina), which were also present in the roots of lycopod sporophytes, but absent from the ungerminated spores and the roots of surrounding herbaceous plants, suggesting high mycorrhizal specificity in Lycopodiaceae. In addition, ungerminated spores were profusely parasitized by chytrid fungi, also present in the surrounding lycopod gametophytes and sporophytes, which might explain the low spore germination rate. Altogether, the requirement of specific mycorrhizal Mucoromycotina fungi and the high prevalence of parasites may explain why Lycopodiaceae are often rare pioneer species in temperate regions, limited to the first stages of ecological succession. This illustrates the primordial roles that belowground microbes play in aboveground plant dynamics.

## INTRODUCTION

**FIGURE 1:**
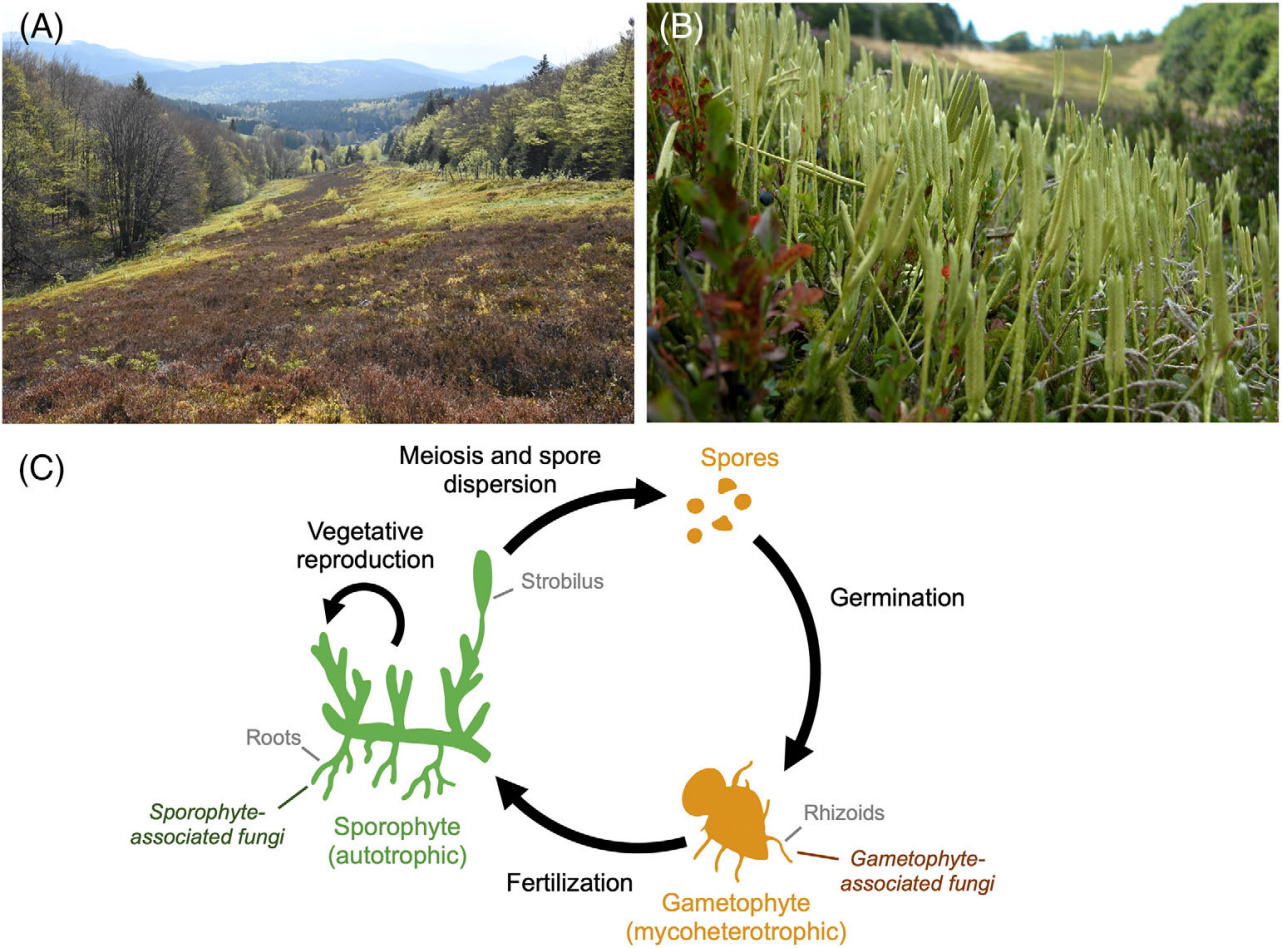
Lycopodiaceae in the Hochfeld reserve (Alsace, France): (A) Photograph of one of the ski trails in the Hochfeld reserve where seven sympatric Lycopodiaceae species have been reported. Trails are surrounded by beech forests where no lycopod has been reported. (B) Photograph of sporophytes of *Lycopodium clavatum* on the ski trail. *Source*: Photo credits: F. Bick and P. Holveck.(C) Representation of the life cycle of Lycopodiaceae, which alternates between a sporophytic stage (autotrophic and producing spores in the strobilus) and a gametophytic stage (after spore germination; generally achlorophyllous, subterranean, and mycoheterotrophic; Boullard, 1979). Haploid stages (spores and gametophyte) are coloured in orange, whereas the diploid stage (sporophyte) is in green. Sporophytic and gametophytic stages are both generally colonized by mycorrhizal fungi. The sporophyte can also spread vegetatively through running stems, as frequently observed in Hochfeld.

The Lycopodiaceae (or clubmoss) is a vascular plant family that emerged more than 350 million years ago. Its ca. 400 known species (Christenhusz & Byng, 2016) colonized many ecosystems and present various life forms, from terrestrial to epiphytic (Øllgaard, 1990), most of which colonize tropical areas. In temperate regions, Lycopodiaceae are generally rare species with patchy distributions and a preference for disturbed communities (García Criado et al., 2017; Kramer & Green, 1990). In Europe, only 17 species of Lycopodiaceae have been reported, of which >20% are experiencing population decline and >35% are reported as nearly threatened in the IUCN Red List (García Criado et al., 2017). Yet, in the Hochfeld reserve (Hohwald‐Zundelkopf forest, Alsace, France), an outstanding total of 7 out of the 17 Lycopodiaceae species present in Europe co‐occur in the same site, thus representing one of the most species‐rich lycopod communities of temperate regions (Boeuf, 2001). These Hochfeld lycopods have emerged only recently following a human‐driven disturbance. Indeed, lycopods were first observed in 1987, at the place where, in the early 1960s, a beech forest was cut and the differentiated soil stripped to build ski trails (Boeuf, 2001; Figure 1A,B). The seven lycopod species therefore thrive on poorly differentiated heathland, with mostly mineral soil, as pioneer species in the early stages of ecological succession. Yet, a decrease in diversity has been observed in the last decades; for example, *Spinulum annotinum* disappeared from the ski trails in Hochfeld, requiring specific conservation measures. This therefore raises the question of the ecological dynamics of Lycopodiaceae in temperate communities and the factors influencing it.

Lycopodiaceae are characterized by the alternation of two free‐living generations: an erected, diploid sporophyte, which is autotrophic, and a haploid gametophyte, which is usually achlorophyllous and subterranean (Boullard, 1979; Figure 1C). Like >90% of land plants, lycopods form mycorrhizal symbioses. At the sporophytic stage, mycorrhizal fungi colonize roots, where organic carbon from plant photosynthesis is exchanged against mineral matter gathered in the soil by the fungi (Hoysted et al., 2019; Smith & Read, 2008). Conversely, many lycopod gametophytes are devoid of photosynthesis and therefore require an external source of organic carbon: they develop an unusual interaction with mycorrhizal fungi, relying on them for both organic and mineral nutrition (Boullard, 1979; Winther & Friedman, 2008), a strategy referred to as ‘mycoheterotrophy’ (Merckx, 2013). In other words, achlorophyllous gametophytes need to interact with mycorrhizal fungi connected with other autotrophic plants that supply organic carbon to the entire system.

As >70% of land plants, lycopods can form arbuscular mycorrhiza with Glomeromycotina (Hoysted et al., 2018; Winther & Friedman, 2008), with which they tend to develop specific interactions, a rather unusual feature compared with other plant–Glomeromycotina interactions (Perez‐Lamarque et al., 2020). In addition, it has been recently reported that some Lycopodiaceae were also colonized by fine non‐septate filamentous fungi from the Endogonales (Mucoromycotina; Perez‐Lamarque, Petrolli, et al., 2022; Rimington et al., 2015) and nutritional exchanges characterizing active mycorrhizae have been demonstrated between lycopods and Mucoromycotina (Hoysted et al., 2019, 2021). Gametophytes and sporophytes tend to share the same fungi (Hoysted et al., 2021; Winther & Friedman, 2008), suggesting that organic carbon could be directly transferred from the autotrophic sporophytes to the mycoheterotrophic gametophytes thanks to a common mycelial network in a kind of parental nurture (Leake et al., 2008). Besides Glomeromycotina and Mucoromycotina, it has been reported that Basidiomycota fungi belonging to the Sebacinales order also often associate with lycopod sporophytes and/or gametophytes (Basidiomycota; Horn et al., 2013; Perez‐Lamarque, Petrolli, et al., 2022; Weiß et al., 2016). Yet, active nutritional exchanges between lycopods and Sebacinales have never been established (Pressel et al., 2016). Sebacinales are also well known to be widespread plant endophytes (sensu Wilson (1995), i.e. fungi that internally colonize plants without major nutritional exchanges; Selosse et al., 2009, 2018; Weiß et al., 2016) or saprotrophs (Weiß et al., 2016). Thus, it remains unclear whether Sebacinales form mycorrhizae with lycopods or if they only colonize them superficially as saprotrophs or internally as endophytes (Rimington et al., 2015; Strullu‐Derrien et al., 2014).

Several hypotheses have been proposed for the rapid emergence of Lycopodiaceae at the early stage of ecological succession on the stripped soil of the Hochfeld reserve (Boeuf, 2001), including the dormancy of lycopod spores in the forest soil (endogenous origin) and the wind dispersal of lycopod spores that could have managed to germinate on the stripped, competitor‐free soil (exogenous origin). In order to investigate the emergence of lycopod gametophytes, we performed a germination experiment in Hochfeld (see Supplementary Methods, S1). In short, we replicated the conditions that enable the emergence of lycopods by implanting spores from four lycopod species (*Diphasiastrum tristachyum* (Pursh) Holub, 1975; *Diphasiastrum oellgaardii* Stoor, Boudrie, Jérôme, K. Horn and Bennert, 1996; *Diphasiastrum zeilleri* (Rouy) Holub, 1975; and *Lycopodium clavatum* L., 1753 subsp. *clavatum*) in a stripped soil nearby lycopod sporophytes on the ski trails. Then, we investigated the spore germination success and the sets of lycopod‐associated fungi that may influence it.

## RESULTS AND DISCUSSION

**FIGURE 2:**
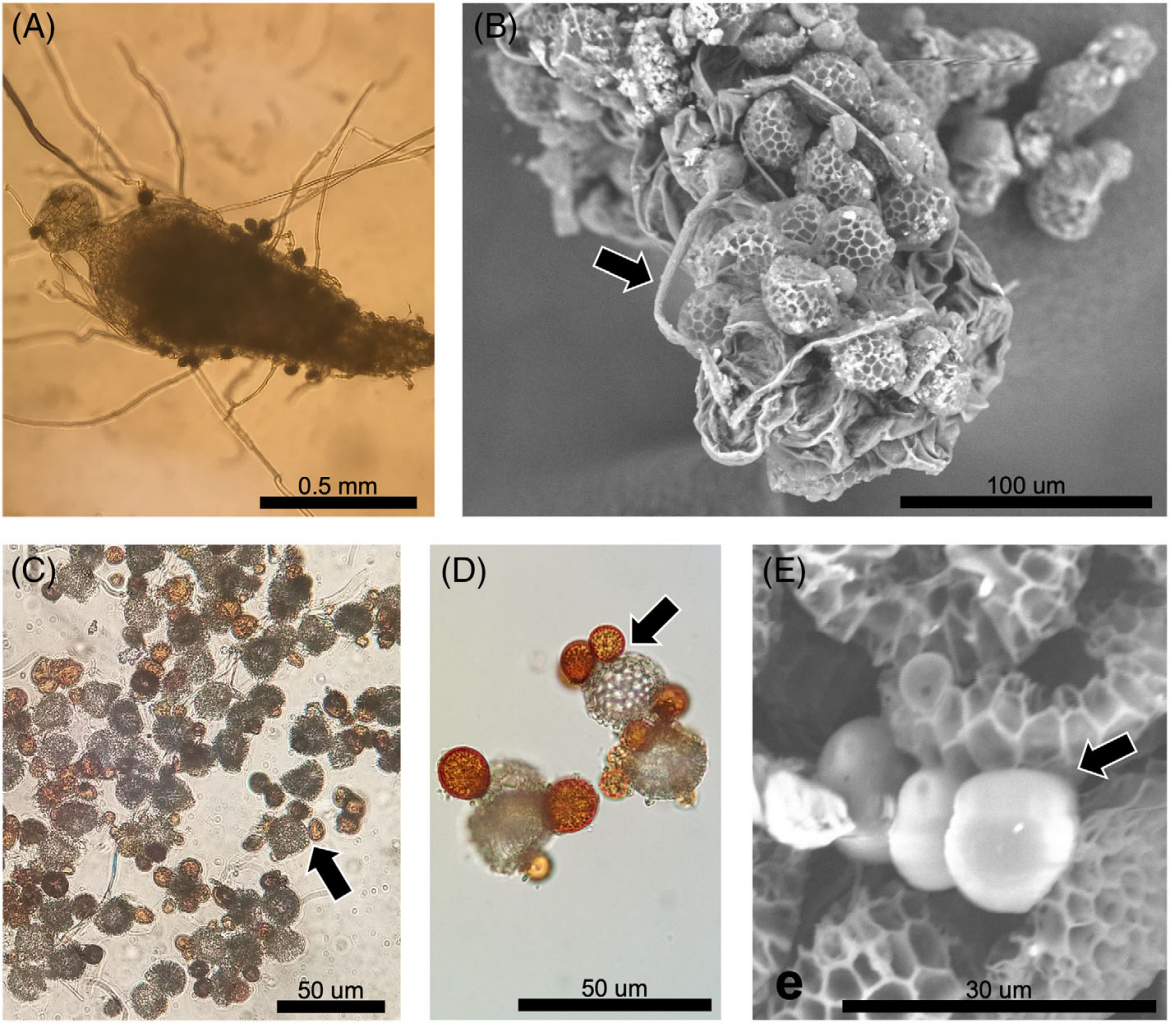
Microscopic observations of a lycopod gametophyte (A, B) and lycopod spores (C). (A) Micrograph of a gametophyte of *Diphasiastrum oellgaardii* using optical microscopy. Black points correspond to ungerminated spores. (A) Micrograph of a gametophyte of *Diphasiastrum zeilleri* using scanning electron microscopy. Several lycopod spores, recognizable by their typical ornamentations, are stuck at the gametophyte surface. Several filaments (e.g. black arrow), which are likely fungal hyphae, surround the gametophyte: they can either correspond to mycorrhizal/endophytic fungi or to saprophytic fungi that colonize it superficially. The wrinkled aspect of the gametophyte is due to its dehydration in the vacuum of the scanning electron microscope. (C–E) Micrograph of ungerminated lycopod spores that are parasitized by chytrid‐like fungi using optical microscopy (C, D) or scanning electron microscopy (E). These fungi form reddish protruding sporocysts (e.g. black arrows) at the surface of the spores (in grey).

Plastic slides containing the initial lycopod spores were extracted from the soil after a period of 2–6 years (Figure S1). Some of the slides (~25%) were partially invaded by the roots of surrounding herbaceous plants. A total of 25 slides (~12% of the total slides) contained between 1 and 10 gametophytes at the time of their extraction from the soil, which had sizes ranging from 0.2 to 1.5 mm for the largest (Figure 2A; Table S1). Time spent in the soil did not significantly impact the spore germination success (Table S2). Spores of the four investigated lycopod species had germinated, although 40% of the harvested gametophytes correspond to *D. oellgaardii*. Using a scanning electron microscope, we observed some filaments surrounding gametophytes that might correspond to fungal hyphae (Figure 2B). Conversely, in the large majority of the slides (~88%), the spores did not germinate and no gametophyte was observed. In addition, microscopic observations revealed that many of these spores present characteristic signs of infections by parasitic chytrid‐like fungi (Figure 2C–E), suggesting that they are no longer viable.

We characterized the fungal communities associated with lycopod gametophytes by metabarcoding of the 18S rRNA gene and the ITS2 region (see Supplementary Methods S2). Besides gametophytes, we comparatively looked at the fungi present (i) in ungerminated spores, (ii) in the roots of herbaceous plants growing inside the plastic slides (i.e. in contact with lycopod spores and/or gametophytes), and (iii) in the roots of surrounding lycopod sporophytes collected close to the experimental plots (within 5–100 m). We successfully amplified fungal DNA from 11 gametophytes among the 15 collected; amplified gametophyte samples belonged to the three *Diphasiastrum* species (Table S1). In addition, we amplified fungal DNA from 28 spore samples, 23 herbaceous roots, and 31 sporophyte roots ( Table S1). A total of 183 operational taxonomic units (OTUs) were detected based on the 18S rRNA gene and 297 OTUs based on the ITS2 region. Because we amplified all fungal DNA present in each sample type, the observed fungi can correspond to active mycorrhizal fungi, to endophytic fungi, or to saprophytic fungi that simply grew in the vicinity and/or the surface of the samples without really interacting with them.

**FIGURE 3:**
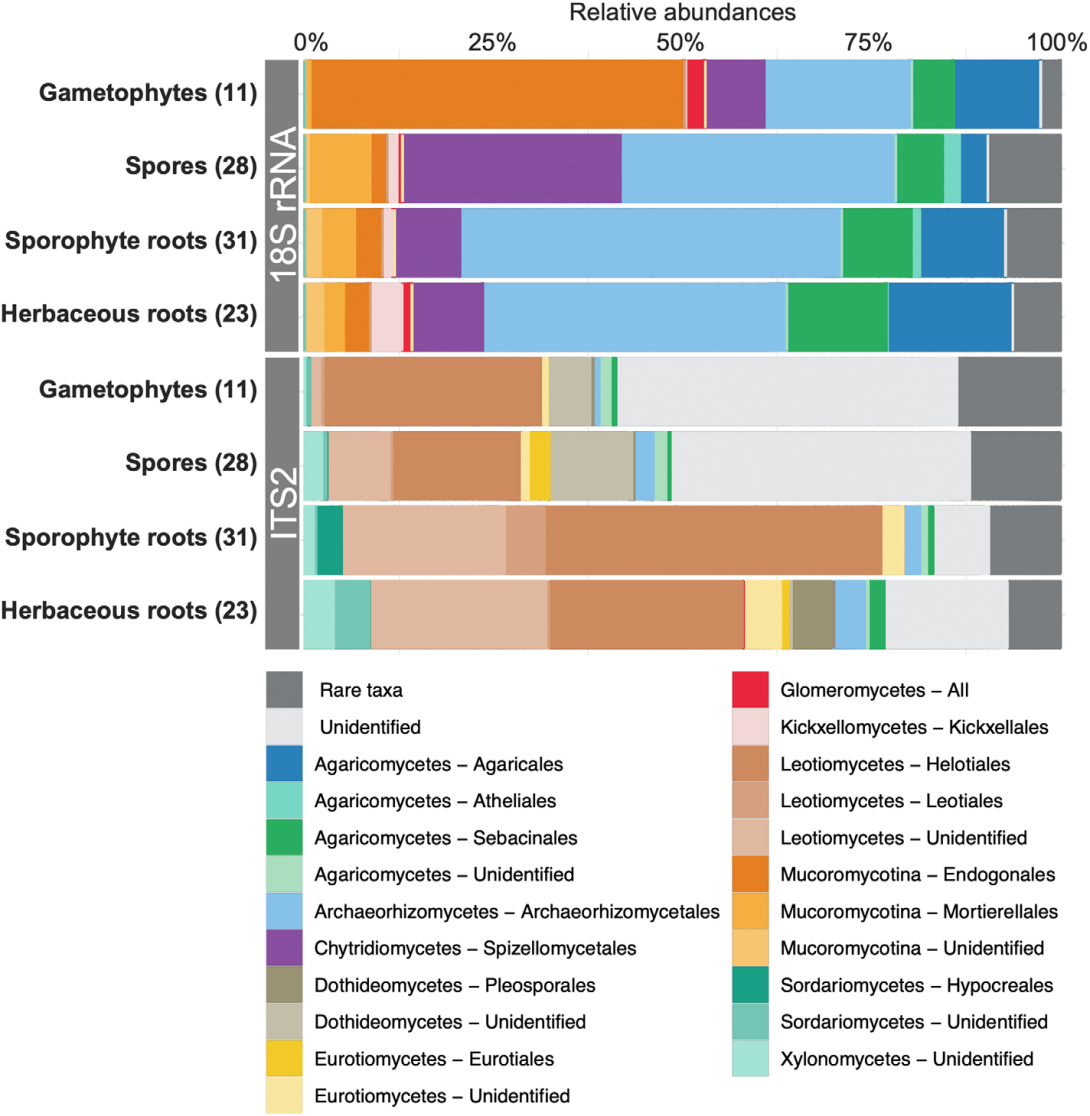
The relative composition of the mycobiomes associated with the different sample types revealed that Lycopodiaceae gametophytes are abundantly colonized by Endogonales (Mucoromycotina) while spores are parasitized by Chytridiomycota. Mycobiome composition in the different samples (lycopod gametophytes, lycopod spores, roots of lycopod sporophytes, or roots of herbaceous plants surrounding the gametophytes) based on 18S rRNA or ITS metabarcoding. For each sample type, the number of individual samples is indicated in brackets. The bar plots represent in colours the class and the order of the main fungal lineages. Rare taxa (representing less than 0.5% of the data) are represented in dark grey. Only results for the Swarm OTUs are represented (analyses based on 97% OTUs gave very similar results; Figure S9).

Investigations based on the combination of both complementary metabarcodes revealed the composition of the mycobiome of these samples (Figure 3). Based on the 18S rRNA barcode, we noticed that the lycopod gametophytes were abundantly colonized by Endogonales (Mucoromycotina; ~50% of the total fungal reads; Figure 3). Endogonales were also present in other sample types, including spores and sporophyte roots, as previously reported in lycopods (Benucci et al., 2020; Hoysted et al., 2019; Perez‐Lamarque, Petrolli, et al., 2022; Rimington et al., 2015). Conversely, Glomeromycotina were only marginally present (<5% of the total fungal reads in gametophytes), confirming that mycorrhizal associations with Glomeromycotina are facultative for some Lycopodiaceae species (Rimington et al., 2015), as long as other mycorrhizal fungi are present. In spore samples, >25% of the fungal reads belong to Spizellomycetaceae (Chytridiomycota), a family of plant‐parasitic fungi (Freeman et al., 2009; Lozupone & Klein, 2002; Powell, 1993) that is likely responsible for the spore degradation we observed (Figure 2C–E). We also detected Chytridiomycota in lower abundances in all other sample types, including gametophytes and sporophytes. Many samples, including gametophytes, were also associated with Sebacinales (Figure S2), confirming that such lycopod‐Sebacinales associations are frequent (Horn et al., 2013; Perez‐Lamarque, Petrolli, et al., 2022). Finally, Archaeorhizomycetes, a major clade of soil saprophytic fungi (Naranjo‐Ortiz & Gabaldón, 2019), was abundantly present in most of the samples with abundances sometimes >50%. Based on the ITS region, we failed to identify taxonomically most of the fungi present in gametophytes and spores, probably because these fungi present in the mineral soil we investigated are under‐represented in ITS taxonomic databases. Among the identified fungi, we nevertheless observed that Helotiales, which are widespread endophytes (Walker et al., 2011), were abundantly associated with most samples, including gametophytes (~30% of the total fungal reads) and sporophytes (~50%) as previously reported in Lycopodiaceae (Benucci et al., 2020) and other plants (Almario et al., 2022). We also detected Sebacinales in low abundances in many samples (Figure S3). However, we did not detect any Mucoromycotina using the ITS marker, indicating that using only the ITS2 region can drastically bias the detection of Mucoromycotina symbionts. Thus, the non‐detection of Mucoromycotina symbionts when using the ITS2 region alone may explain the detection of only Sebacinales in the gametophytes of *Diphasiastrum alpinum* by Horn et al. (2013) (Strullu‐Derrien et al., 2014). Using 18S rRNA and ITS2 metabarcoding in combination is thus required to better characterize lycopod‐associated mycobiomes.

We observed that the types of samples significantly impacted the composition of the fungal communities characterized using 18S (PermANOVA: *R* = 0.09, *p* < 0.001) or ITS (*R* = 0.13, p < 0.001; Figures S2–S4). In addition, fungal communities were also influenced to a lesser extent by the lycopod species (18S: *R* = 0.07, *p* = 0.01; ITS: *R* = 0.09, *p* < 0.001). This was observed in the PCoA plots and interaction networks where samples primarily cluster per sample type and then per species (Figure S4). Species‐specific fungal communities have already been demonstrated in the roots of lycopod sporophytes (Benucci et al., 2020), but we additionally showed here that they also vary significantly in gametophytes and spores of different Lycopodiaceae species. Significant differences across sample types indicate that lycopods are thus associated with (partially) different fungal communities during their life cycles, similarly to what is often observed in orchids, which are also mycoheterotrophic during germination (Bidartondo & Read, 2008; Waud et al., 2017).

**FIGURE 4:**
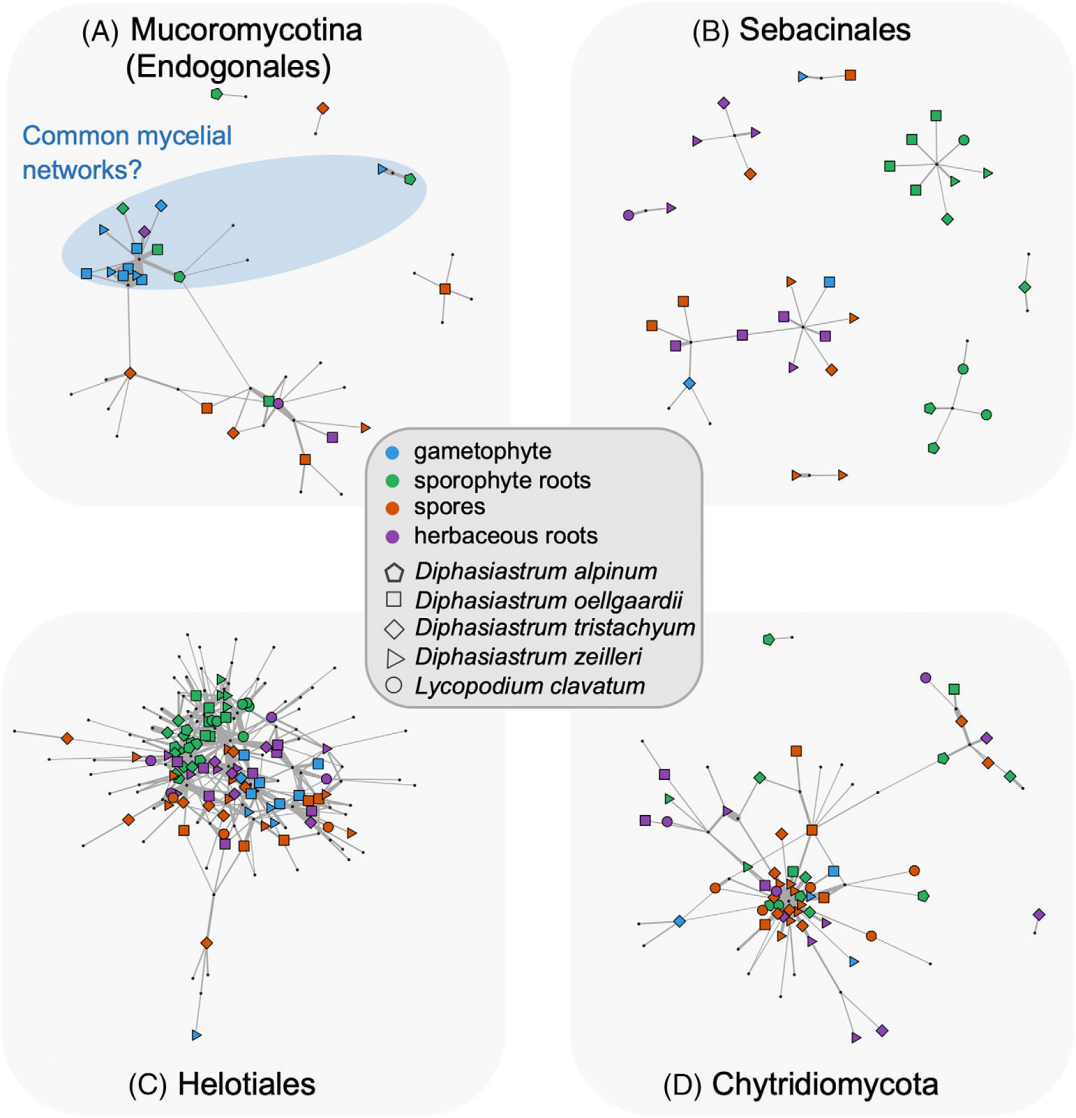
Plant–fungus association networks reveal that fungal OTU sharing between plant samples is frequent for Endogonales and Chytridiomycota, but less so for Sebacinales and Helotiales: Network representation at the sample level for the different fungal groups: (A) (Mucoromycotina [Endogonales]), (B) Sebacinales, (C) Helotiales, or (D) Chytridiomycota. Large coloured nodes represent individual samples (colours indicate the different types of samples while shapes indicate the different lycopod species) and small grey nodes correspond to fungal OTUs. Grey links represent plant–fungus associations and their widths are proportional to their abundances. The position of the nodes reflects the similarity in species associations using the Fruchterman–Reingold layout algorithm (Fruchterman & Reingold, 1991). Networks were visualized using the *igraph* R‐package for the Swarm OTUs (analyses based on 97% OTUs gave very similar results). We emphasize in blue the sharing of Mucoromycotina OTUs between Lycopodiaceae gametophytes and sporophytes that may correspond to common mycorrhizal networks linking adults and germinations.

**FIGURE 5:**
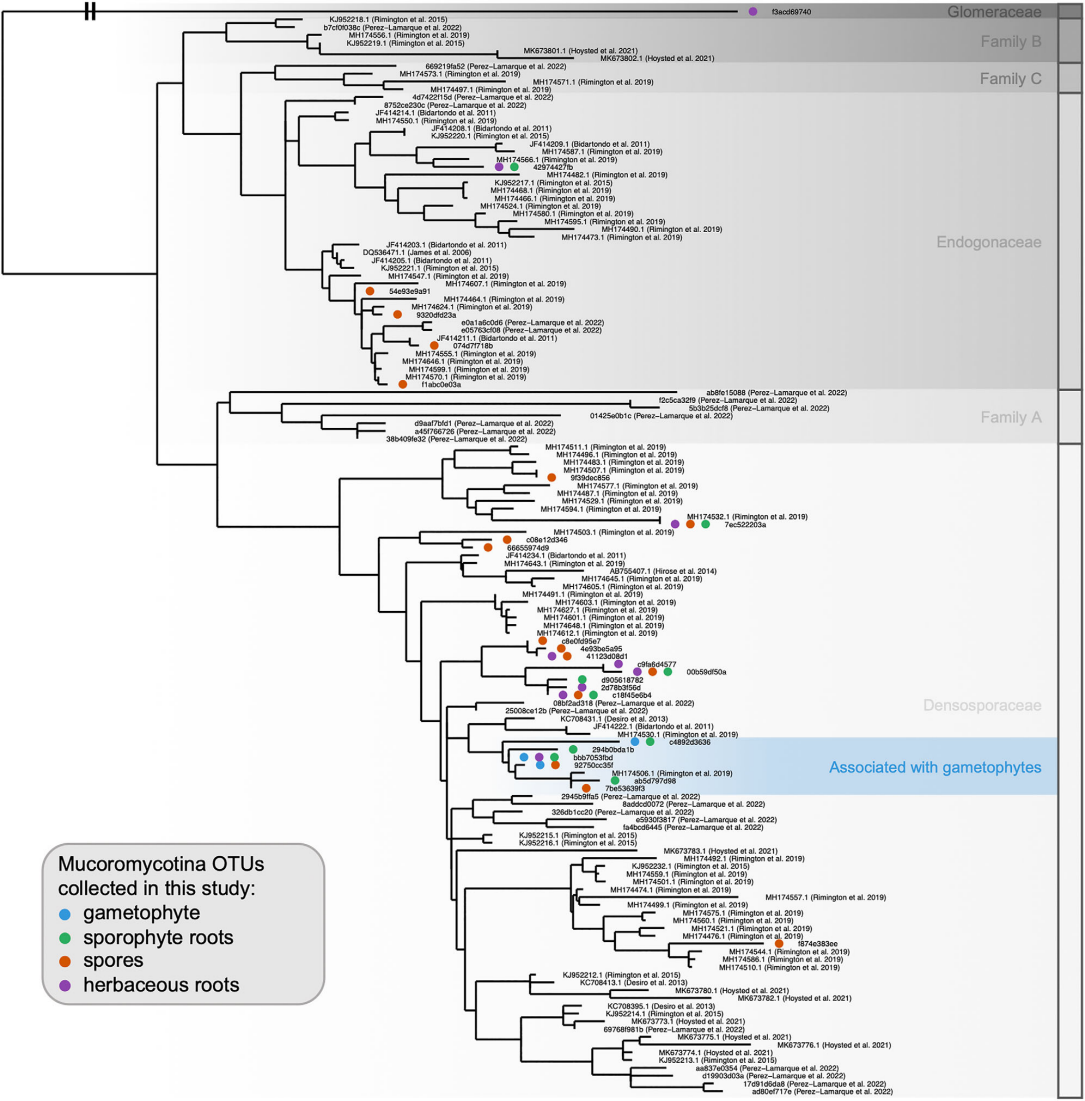
Gametophytes and sporophytes of Lycopodiaceae associate with a specific clade of Densosporaceae: Maximum‐likelihood phylogenetic tree of the Endogonales (Mucoromycotina) including the 18S rRNA OTUs detected in this study, as well as reference Endogonales sequences from previous studies looking at Endogonales colonization in lycopods or other early‐diverging plant lineages (Hoysted et al., 2021; Perez‐Lamarque, Petrolli, et al., 2022; Rimington et al., 2015, 2019). The origin of each sample is indicated in brackets, with the indication of the type of samples (lycopod gametophytes, lycopod spores, roots of lycopod sporophytes, or roots of herbaceous plants surrounding the gametophytes) where each Endogonales OTU was observed. The taxonomy of Endogonales, including the families Endogonaceae, Densosporaceae, and the families ‘A’', ‘B’', and ‘C’', follows the designation of Rimington et al. (2019). This phylogeny matches the Endogonales multigene phylogeny obtained by Desirò et al. (2017).

Then, we investigated whether OTUs from the main fungal lineages were ‘shared’ between samples using network visualization. Thereby, we built plant–fungus interaction networks for the Endogonales (using the 18S barcode), the Sebacinales (using both ITS and 18S barcodes), the Helotiales (ITS barcode), and the Chytridiomycota (18S barcode; see Supplementary Methods S3). For the Endogonales (Mucoromycotina), we first reported that both gametophytes and sporophytes were only interacting with a limited number of specific OTUs, irrespective of the lycopod species (PermANOVA: *R* = 0.16, *p* = 0.33; Figure 4A; Figure S5). Indeed, only three Mucoromycotina OTUs colonized lycopod gametophytes, which all belong to Densosporaceae and form a separate clade of closely related fungi (Figure 5; Desirò et al., 2017). Such Densosporaceae have already been found to form mycorrhizae in lycopods (Hoysted et al., 2019; Rimington et al., 2015), and the closest relative of these gametophyte‐associated Densosporaceae OTUs has been observed in liverworts (in *Fossombronia foveolata*; Rimington et al., 2019; Figure 5). Thorough Blast analyses on GenBank confirmed that members of this Densosporaceae clade have (currently) only been reported to associate with lycopods or other early‐diverging plant lineages. Although more gametophytes sampled in different locations are required to confirm or not the specificity of these interactions with Densosporaceae, the fact that Densosporaceae OTUs are (only) shared between gametophytes and sporophytes suggests that they may form common mycelial networks. Such a high specificity has already been proposed for lycopod–Glomeromycotina interactions (Perez‐Lamarque et al., 2020; Winther & Friedman, 2008). Future work should investigate the prevalence of mycorrhizal specificity in lycopod–fungus interactions and test (e.g. by isotopic labelling) whether or not the resulting mycelial networks sustain the gametophytes by providing resources derived from surrounding adults and thus promote spore germination (i.e. parental nurture; Leake et al., 2008).

Lycopod sporophytes also interact with other Endogonales, including Endogonaceae (Figure 4A; Figure S5). Some of these Endogonales OTUs were also detected in herbaceous roots, confirming that lycopod sporophytes and angiosperms may share the same Mucoromycotina symbionts (Hoysted et al., 2019). Yet, most of the OTUs detected in lycopod spores and in herbaceous roots were not observed in lycopod gametophytes or sporophytes (Figure 4; Figure S5). This may suggest that Mucoromycotina OTUs only associated with ungerminated spores and herbaceous roots may not be able to form mycorrhizae with Lycopodiaceae. Indeed, many Endogonales have retained saprophytic abilities (Chang et al., 2019). Therefore, the absence of specific mycorrhizal OTUs could partially explain why spores often did not germinate. Isolating and culturing the Densosporaceae fungi to perform in vitro germination of Lycopodiaceae will be challenging (Whittier, 1977) but would provide a better understanding of the functioning of Densosporaceae‐lycopod associations and be particularly valuable for conservation purposes.

Our results therefore tend to confirm the pervasiveness and importance of Mucoromycotina in lycopods (Hoysted et al., 2019; Perez‐Lamarque, Petrolli, et al., 2022; Rimington et al., 2015), as in many other vascular or non‐vascular plant lineages (Desirò et al., 2013; Hoysted et al., 2018; Rimington et al., 2019; Sinanaj et al., 2021). Finding frequent associations with Mucoromycotina (and infrequent associations with Glomeromycotina) in the heathland of the Hochfeld reserve, which is likely deprived of nitrogen, may be explained by the important contribution of Mucoromycotina to plant nitrogen nutrition (Howard et al., 2022; Hoysted et al., 2019). Further work is needed to investigate the prevalence of Mucoromycotina in particular environments, such as nitrogen‐deprived ones (Perez‐Lamarque, Petrolli, et al., 2022).

In contrast with the sharing of Mucoromycotina OTUs between sporophytes and gametophytes, the Sebacinales OTUs were markedly different between sporophytes and gametophytes (Figure 4B; Figure S6). Instead, Sebacinales OTUs tend to be rather specific to the different sample types and lycopod species (PermANOVA: sample types: *R* = 0.22, *p* < 0.001; species: *R* = 0.15, *p* = 0.01; SFigure S6). Similar patterns were observed when using the 18S rRNA barcode instead of the ITS barcode to characterize Sebacinales (Figure S6). Like the Sebacinales, Helotiales OTUs were significantly different between sample types and lycopod species (Figure 4C; Figure S7). In particular, we noted very little OTU sharing between sporophytes and gametophytes, or with the surrounding herbaceous plants. Therefore, we suspect that these fungal groups are unlikely to sustain gametophytes with organic carbon through common mycelial networks (i.e. mycoheterotrophy) and are thus probably just saprotrophic and/or endophytic fungi (Pressel et al., 2016; Selosse et al., 2009; Walker et al., 2011) with limited relevance. Nevertheless, to properly test whether or not these fungal lineages form mycorrhizal symbioses with lycopods, future studies should include fine‐scale microscopic observations and isotope experiments specifically targeting Sebacinales or Helotiales (as, e.g. Hoysted et al., 2021; Schneider‐Maunoury et al., 2020).

Finally, for Chytridiomycota, the majority of the samples were colonized by a single and abundant OTU from the *Spizellomyces* genus (Figure 4D). This parasitic fungus, frequently found degrading pollen and other plant tissues (Lozupone & Klein, 2002), was particularly frequent in lycopod spores, but also detected in lycopod gametophytes and sporophytes, and it did not show any specificity towards any lycopod species (*R* = 0.07, *p* = 0.54; Figure S8). Therefore, spore parasitism preventing their germination seems to be mainly explained by one dominant generalist OTU abundantly present in all developmental stages of the Lycopodiaceae species collected in the Hochfeld reserve.

Experimental germinations in the Hochfeld reserve confirmed the pervasiveness of Mucoromycotina as mycorrhizal symbionts of Lycopodiaceae and suggested that lycopod spores require specific associations with Densosporaceae to germinate and sustain gametophytes. In addition, we found that spores of all Lycopodiaceae species in this community were heavily parasitized by *Spizellomyces* (Chytridiomycota). Therefore, both the limitation in availability of lycopod‐compatible mycorrhizal fungi and the presence of fungal parasites could explain the low germination success of the spores we observed. Going back to the different hypotheses explaining the emergence of seven Lycopodiaceae species in the Hochfeld reserve after disturbance, we suspect that spore dormancy (i.e. an endogenous origin) is unlikely since we found a fast degradation of lycopod spores in the soil. In contrast, an exogenous origin by wind dispersal is more likely. Given that lycopod gametophytes tend to require specific mycorrhizal fungi, which do not seem to be widespread in surrounding plant roots in the Hochfeld reserve, the establishment of Lycopodiaceae would require the simultaneous arrival of lycopod and Densosporaceae spores, which are both small and likely to be efficiently wind‐dispersed (Vittoz & Engler, 2007). Our findings therefore fit with the idea that many Lycopodiaceae species are pioneer species following a disturbance (García Criado et al., 2017; Kramer & Green, 1990). Thanks to efficient spore dispersal, Lycopodiaceae may rapidly colonize a disturbed site during the early stage of ecological succession. Yet, at later stages, the accumulation of specific parasites, like the chytrid fungi that degrade lycopod spores, likely affect their reproduction and survival and may drive their local extinction (Van der Putten et al., 1993), as reported for *Lycopodium annotinum* in the Hochfeld reserve. Indeed, there is generally a trade‐off between investing in defences against pathogens and rapidly settling in a community after a disturbance. The accumulation of pathogens over time thus often drives the decline of pioneer plant species such as lycopods (Bever et al., 2015; Van der Putten et al., 1993). Altogether, this illustrates the primordial roles that belowground mutualistic and parasitic microbes play in aboveground plant dynamics during ecological succession (Wardle et al., 2004). Considering such plant–soil feedback will be particularly critical for future conservation planning.

## AUTHOR CONTRIBUTIONS

All authors designed the study. Didier Epp, François Labolle, Francis Bik, Pascal Holveck, and Marc‐André Selosse started the experiments. Didier Epp, François Labolle, Francis Bik, Pascal Holveck, Benoît Perez‐Lamarque, Liam Laurent‐Webb, and Marc‐André Selosse collected and processed the samples in the field. Benoît Perez‐Lamarque, Amélia Bourceret, and Louis Maillet performed the molecular work. Benoît Perez‐Lamarque performed the statistical analyses and wrote the first version of the manuscript. All authors contributed to the revisions.

## CONFLICT OF INTEREST

The authors declare no conflict of interest.

## Supporting information


**Supplementary Methods 1:** Experimental design.
**Supplementary Methods 2:** Molecular analyses and bioinformatics.
**Supplementary Methods 3:** Statistical analyses.
**Supplementary Figure 1:** Experimental design in the Hochfeld reserve: (a) Representation of the organization of each experimental plot: Points indicate where the plastic slides containing lycopod spores were sowed in the soil. The different shapes represent the different dates of implantation in the soil, and the colours indicate the four lycopod species. Crosses represent the dates of extractions of the slides from the soil. All the remaining slides were extracted in May 2021. (b–d) Photographs of one experimental plot: at the beginning of the experiment (after the mechanical stripping of the soil); (b) and in autumn 2018 (d). (c) Present a plastic slide containing spores of *Lycopodium clavatum* before being sowed in the soil. Photo credits: P. Holveck.
**Supplementary Figure 2:** Mycobiome composition based on 18S rRNA metabarcoding:
Mycobiome composition in the different samples (lycopod gametophytes, lycopod spores, roots of lycopod sporophytes, or roots of herbaceous plants surrounding the gametophytes) and different lycopod species (*Lycopodium clavatum*, *Diphasiastrum tristachyum*, *Diphasiastrum oellgaardii*, *Diphasiastrum zeilleri*, and *Diphasiastrum alpinum*) based on 18S rRNA metabarcoding. For each sample type and each lycopod species, the number of individual samples is indicated in brackets. The bar plots represent in colours the class and the order of the main fungal lineages. Rare taxa (representing less than 0.5% of the data are represented in dark grey). Only results for the Swarm OTUs are represented (analyses based on 97% OTUs gave very similar results).
**Supplementary Figure 3:** Mycobiome composition based on ITS2 metabarcoding: Mycobiome composition in the different samples (lycopod gametophytes, lycopod spores, roots of lycopod sporophytes, or roots of herbaceous plants surrounding the gametophytes) and different lycopod species (*Lycopodium clavatum*, *Diphasiastrum tristachyum*, *Diphasiastrum oellgaardii*, *Diphasiastrum zeilleri*, and *Diphasiastrum alpinum*) based on ITS2 metabarcoding. For each sample type and each lycopod species, the number of individual samples is indicated in brackets. The bar plots represent in colours the class and the order of the main fungal lineages. Rare taxa (representing less than 0.5% of the data are represented in dark grey). Only results for the Swarm OTUs are represented (analyses based on 97% OTUs gave very similar results).
**Supplementary Figure 4:** Mycobiome composition significantly varies across sample types and lycopod species: (a, b) Principal coordinate analyses (PCoA) of the mycobiomes characterized based on ITS2 (a) or 18S rRNA (b) metabarcoding. Each panel represents the projection of all the samples onto the two first axes of the PCoA performed on Bray–Curtis dissimilarities. Each sample is coloured according to the sample type, while the shape indicates the lycopod species. The results of PermANOVA testing for the effect of sample type or lycopod species on mycobiome composition are indicated at the top of the panel. (c, d) Plant–fungus interaction networks at the sample‐level characterized based on ITS2 (c) or 18S rRNA (d) metabarcoding. Large coloured nodes represent individual samples and small grey nodes correspond to fungal OTUs. Grey links represent plant–fungus interactions and their widths are proportional to interaction abundances. The position of the nodes reflects the similarity in species interactions using the Fruchterman–Reingold layout algorithm (Fruchterman and Reingold, 1991).
**Supplementary Figure 5:** Mucoromycotina OTUs are shared between lycopod gametophytes and sporophytes: (a) Principal coordinate analyses (PCoA) of the sets of Mucoromycotina OTUs associated with each sample characterized using 18S rRNA metabarcoding. It represents the projection of all the samples onto the two first axes of the PCoA performed on Bray–Curtis dissimilarities. Each sample is coloured according to the sample type, while the shape indicates the different lycopod species. The results of PermANOVA testing for the effect of sample type or lycopod species on Mucoromycotina composition are indicated at the top of the panel. (b) Venn diagram of the Mucoromycotina OTUs between gametophytes of different lycopod species: Mucoromycotina OTUs are largely shared between gametophytes of different species. (c) Venn diagram of the Mucoromycotina OTUs between different sample types: Mucoromycotina OTUs are largely shared between gametophytes and sporophytes, but the Mucoromycotina OTUs found in samples of spores or herbaceous roots almost never colonize gametophytes.
**Supplementary Figure 6:** Sebacinales OTUs are poorly shared between sample types and lycopod species: (a) Principal coordinate analyses (PCoA) of the sets of Sebacinales OTUs associated with each sample characterized using ITS metabarcoding. It represents the projection of all the samples onto the two first axes of the PCoA performed on Bray–Curtis dissimilarities. Each sample is coloured according to the sample type, while the shape indicates the different lycopod species. The results of PermANOVA testing for the effect of sample type or lycopod species on Sebacinales composition are indicated at the top of the panel. (b) Venn diagram of the Sebacinales OTUs between gametophytes of different lycopod species: Sebacinales OTUs are not shared between gametophytes of different species. (c) Venn diagram of the Sebacinales OTUs between different sample types: Many Sebacinales OTUs are not shared between samples from different types. (d) Plant–Sebacinales interaction network at the sample‐level characterized using 18S rRNA metabarcoding. Large coloured nodes represent individual samples and small grey nodes correspond to fungal OTUs. Grey links represent plant–fungus interactions and their widths are proportional to interaction abundances. The position of the nodes reflects the similarity in species interactions using the Fruchterman–Reingold layout algorithm (Fruchterman and Reingold, 1991).
**Supplementary Figure 7:** Helotiales OTUs are poorly shared between sample types and lycopod species: (a) Principal coordinate analyses (PCoA) of the sets of Helotiales OTUs associated with each sample characterized using ITS metabarcoding. It represents the projection of all the samples onto the two first axes of the PCoA performed on Bray–Curtis dissimilarities. Each sample is coloured according to the sample type, while the shape indicates the different lycopod species. The results of PermANOVA testing for the effect of sample type or lycopod species on Helotiales composition are indicated at the top of the panel. (b) Venn diagram of the Helotiales OTUs between gametophytes of different lycopod species: Helotiales OTUs are poorly shared between gametophytes of different species. (c) Venn diagram of the Helotiales OTUs between different sample types: Many Helotiales OTUs are not shared between samples from different types.
**Supplementary Figure 8:** One Chytridiomycota OTU is largely shared between sample types and lycopod species: (a) Principal coordinate analyses (PCoA) of the sets of Chytridiomycota OTUs associated with each sample characterized using 18S rRNA metabarcoding. It represents the projection of all the samples onto the two first axes of the PCoA performed on Bray–Curtis dissimilarities. Each sample is coloured according to the sample type, while the shape indicates the different lycopod species. The results of PermANOVA testing for the effect of sample type or lycopod species on Chytridiomycota composition are indicated at the top of the panel. (b) Venn diagram of the Chytridiomycota OTUs between gametophytes of different lycopod species: one Chytridiomycota OTUs is shared between all lycopod species. (c) Venn diagram of the Chytridiomycota OTUs between different sample types: Chytridiomycota OTUs are quite shared sample types and are especially abundant in spores.
**Supplementary Figure 9:** When using 97% OTU clustering instead of Swarm clustering, the relative composition of the mycobiomes associated with the different sample types also revealed that Lycopodiaceae gametophytes are abundantly colonized by Endogonales (Mucoromycotina) while spores are parasitized by Chytridiomycota. Mycobiome composition in the different samples (lycopod gametophytes, lycopod spores, roots of lycopod sporophytes, or roots of herbaceous plants surrounding the gametophytes) based on 18S rRNA or ITS metabarcoding. For each sample type, the number of individual samples is indicated in brackets. The bar plots represent in colours the class and the order of the main fungal lineages. Rare taxa (representing less than 0.5% of the data are represented in dark grey). Only results for the 97% OTUs are represented here; analyses based on Swarm OTUs gave very similar results (see Figure 3).
**Supplementary Table 1:** Gametophytes were present in 25 plastic slides: This table recapitulates the gametophyte germinations and the different samples that were collected. The first column indicates the number of slides where lycopod gametophytes were observed (among a total of 216 slides, 54 per lycopod species). The second column indicates the number of samples that were collected (if several gametophytes were present in the same slide, they were pooled in the same samples); in addition, we indicated the number of spore samples that were collected, the number of root samples of herbaceous plants (that entered in the slides), and the number of root samples of lycopod sporophytes that were collected in the neighbourhood of the experimental plots. The last column indicates the number of samples for which we successfully managed to amplify fungi using metabarcoding. A few gametophyte samples failed at amplifying fungi, probably because of their very small size (<0.3 mm).
**Supplementary Table 2:** Number of gametophytes observed at the different extractions: Click here for additional data file.

## Data Availability

Raw sequences and associated metadata are available in the Sequence Read Archive (SRA) under the BioProject accession no. PRJNA837378. Scripts used for generating the OTU tables are available at https://github.com/BPerezLamarque/Scripts following Perez‐Lamarque, Krehenwinkel, et al. (2022).
