# Supplementary material for: Fungal microbiomes associated with Lycopodiaceae during ecological succession
Source: Environ Microbiol Rep. 2022 Oct 10;15(2):109–18. doi: 10.1111/1758-2229.13130 (PMC10103886; doi:10.1111/1758-2229.13130)
Supplement: Supplementary file 1 — Supplementary Methods 1: Experimental design. Supplementary Methods 2: Molecular analyses and bioinformatics. Supplementary Methods 3: Statistical analyses. Supplementary Figure 1: Experimental design in the Hochfeld reserve: (a) Representation of the organization of each experimental plot: Points indicate where the plastic slides containing lycopod spores were sowed in the soil. The different shapes represent the different dates of implantation in the soil, and the colours indicate the four lycopod species. Crosses represent the dates of extractions of the slides from the soil. All the remaining slides were extracted in May 2021. (b–d) Photographs of one experimental plot: at the beginning of the experiment (after the mechanical stripping of the soil); (b) and in autumn 2018 (d). (c) Present a plastic slide containing spores of Lycopodium clavatum before being sowed in the soil. Photo credits: P. Holveck. Supplementary Figure 2: Mycobiome composition based on 18S rRNA metabarcoding: Mycobiome composition in the different samples (lycopod gametophytes, lycopod spores, roots of lycopod sporophytes, or roots of herbaceous plants surrounding the gametophytes) and different lycopod species (Lycopodium clavatum, Diphasiastrum tristachyum, Diphasiastrum oellgaardii, Diphasiastrum zeilleri, and Diphasiastrum alpinum) based on 18S rRNA metabarcoding. For each sample type and each lycopod species, the number of individual samples is indicated in brackets. The bar plots represent in colours the class and the order of the main fungal lineages. Rare taxa (representing less than 0.5% of the data are represented in dark grey). Only results for the Swarm OTUs are represented (analyses based on 97% OTUs gave very similar results). Supplementary Figure 3: Mycobiome composition based on ITS2 metabarcoding: Mycobiome composition in the different samples (lycopod gametophytes, lycopod spores, roots of lycopod sporophytes, or roots of herbaceous plants surrounding the gametophytes) and [file EMI4-15-109-s001.pdf]

Supplementary Information

Fungal microbiomes associated with Lycopodiaceae during ecological succession

Benoît Perez-Lamarque, Liam Laurent-Webb, Amélia Bourceret,
Louis Maillet, Francis Bik, Denis Cartier, François Labolle, Pascal Holveck,
Didier Epp, Marc-André Selosse

Supplementary Methods:

Supplementary Methods 1: Experimental design:

The study was conducted on the Hochfeld national biological reserve in the Hohwald-Zundelkopf forest (Alsace, France) between 2012 and 2021 (GPS: N48°24′07′′ E7°16′02′′). The reserve has an average elevation of 945 meters and is characterized by continental but wet weather (average temperature 6.8°C and annual precipitation of 1,544 mm) including 2 to 3 months of snow cover (Durr-Ecolor, 2021). Originally, the reserve was composed of a beech forest, in which a ski resort station was created in the early 1960s (Boeuf, 2001). The forest had been partially cut to make two ski trails and the differentiated soils of the forest were stripped. Nowadays, the ski trails are thus composed of poorly differentiated granite, forming a subalpine heathland, colonized by herbaceous plants, bryophytes, lichens, and lycopods (Durr-Ecolor, 2021). Lycopod sporophytes were first described in 1987 (Boeuf, 2001), more than 20 years after the disturbance. The site originally hosted 7 lycopod species (Supplementary Figure 10): *Lycopodium clavatum* (L.) 1753 subsp. *clavatum*, *Diphasiastrum alpinum* (L.) Holub, 1975, *Diphasiastrum tristachyum* (Pursh) Holub, 1975,

Diphasiastrum × *oellgaardii* Stoor, Boudrie, Jérôme, K.Horn & Bennert, 1996, *Diphasiastrum* × *zeilleri* (Rouy) Holub, 1975, *Spinulum annotinum* (L.) A. Haines, 2003 subsp. *annotinum*, *Huperzia selago* (L.) Bernh. Ex Schrank & Mart., 1829 subsp. *selago.* Yet, *Spinulum annotinum* has not been present on the ski trails since 2001 and *Huperzia selago* is almost absent, suggesting that the diversity of the site is decreasing.

To perform our germination experiment, six plots of 9m^2 were mechanically stripped with a mini-excavator to a depth of 10 centimeters in November 2012 to reproduce the conditions where the founder lycopod spores germinated. Lycopod spores were collected in September 2014 for 4 different lycopod species (*Diphasiastrum tristachyum, Diphasiastrum oellgaardii, Diphasiastrum zeilleri,* and *Lycopodium clavatum*) and put in plastic slides surrounded by plankton net with a mesh of 20 µm, such that spores could not exit the slides, but water and fungal hyphae could go in and out. 96 slides (16 per plot) were sowed in October 2014, 72 slides (12 per plot) in October 2015, and 48 (8 per plot) in November 2016 (Supplementary Figure 1). 24 slides were extracted in September 2015, 24 slides in September 2016, 48 slides in September 2017, 24 slides in October 2019, and all the remaining slides were finally extracted in May 2021. Plastic slides extracted from the soil were immediately stored in individual plastic bags in a cooler. We also collected some roots of lycopod sporophytes surrounding the experimental plots in May 2021. We harvested the roots of up to 7 individuals per plant species (6 *Lycopodium clavatum, 6 Diphasiastrum oellgaardii, 7 Diphasiastrum tristachyum, 5 Diphasiastrum zeilleri,* and 7 *Diphasiastrum alpinum*), carefully cleaned them with sterile water, and immediately dried them in silica gel.

Back in the lab, the plastic slides were opened under a binocular, in sterile conditions, only up to a few hours after their extraction from the soil. If present, gametophytes were collected for microscopic observations and/or stored in 70% ethanol for molecular characterization of the associated fungi. If several gametophytes were present in the same slides, they were pooled together as the same sample for

molecular analyses. We also collected some ungerminated spores in 28 slides selected at random as well as 23 root samples of some herbaceous plants that managed to enter inside the slides (and were therefore in contact with gametophytes and/or ungerminated spores). We performed microscopic observations of some gametophytes and spores using both optical microscopy and scanning electron microscopy.

Supplementary Methods 2: Molecular analyses and bioinformatics

Samples stored in ethanol, including gametophytes, were rinsed using sterile water before starting DNA extractions. For dried roots, 30 mg of tissue was crushed using sterile tungsten beads in the TissueLyser II (Qiagen). DNA was extracted using the DNeasy Plant Mini kit (Qiagen) following the manufacturer's instructions. Following Perez-Lamarque *et al.* (2022), we characterized the whole fungal diversity by amplifying both the 18S rRNA gene and the ITS2 region, using the tagged primer pairs AMADf-AMDGr (Berruti *et al.*, 2017) and ITS86F-ITS4 (White *et al.*, 1990; Turenne *et al.*, 1999). AMADf-AMDGr can detect various fungal lineages including 'early-diverging' ones, whereas ITS86F-ITS4 is more specific to Ascomycota and Basidiomycota. Amplicon pools were carried out as in Taberlet *et al.* (2018) and Petrolli *et al.* (2021) and sequenced using Illumina 2x250 bp MiSeq technology. Negative controls were carefully included during DNA extraction (Zinger *et al.*, 2019).

The sequencing results were processed using VSEARCH (Rognes *et al.*, 2016) with a pipeline available in GitHub (https://github.com/BPerezLamarque/Scripts/) following Perez-Lamarque, Krehenwinkel, *et al.* (2022). Paired-end reads were assembled, quality checked, demultiplexed with cutadapt (Martin, 2011), and clustered into operational taxonomic units (OTUs) using Swarm (Mahé *et al.*, 2015), a clustering approach based on local thresholds and amplicon abundances. Chimeras

were removed *de novo* and we assigned taxonomy to the OTUs using Silva and UNITE databases (Quast *et al.*, 2013; Nilsson *et al.*, 2019). We filtered out the contaminants of the OTU tables using the decontam pipeline (Davis *et al.*, 2018). Non-fungal OTUs were then discarded for subsequent analyses. The few samples having fewer than 10 reads were discarded in the following analyses. In addition, we also performed a classical 97% OTU clustering using VSEARCH. Yet, because they gave qualitatively similar results, only the results obtained with Swarm are presented in the main text.

We next reconstructed a phylogenetic tree of the Endogonales (Mucoromycotina) OTUs. We also added reference sequences of Endogonales from previous studies looking at plant-Mucoromycotina associations in various plant lineages including lycopods (Rimington *et al.*, 2015, 2018; Hoysted *et al.*, 2021; Perez-Lamarque, Petrolli, *et al.*, 2022). We aligned the DNA sequences using MAFFT (Katoh and Standley, 2013), trimmed the aligned sequences with trimAl (Capella-Gutierrez *et al.*, 2009), selected the best substitution model using ModelFinder (Kalyaanamoorthy *et al.*, 2017), and reconstrued the maximum-likelihood tree using IQ-TREE (Nguyen *et al.*, 2015).

Supplementary Methods 3: Statistical analyses:

To assess whether different sample types and different lycopod species were associated with different fungi, we performed PCoA (principal coordinate analysis) and PermANOVA (permutational analysis of variance) from Bray-Curtis dissimilarity matrices between pairs of samples. The PCoA enables the visualization of differences in composition of the fungal communities per sample. PermANOVA tests whether samples of different types and/or different species tend to have significantly different fungal compositions. It gives the fraction (R) of the variance that can be explained by the sample type and/or by the lycopod species. We performed PermANOVA using the

adonis function from the R-package vegan (Oksanen *et al.*, 2016) with 10,000 permutations.

Then, to investigate whether the fungal OTUs were shared or not between different samples, we built plant-fungus interaction networks. We considered an association occurs between a plant sample and a fungus if the fungal OTU is represented by at least 1% of the total fungal reads of the sample, following the approach of Toju *et al.* (2014). By using a threshold of 1%, we thus corrected the heterogeneous number of reads per sample and avoided counting cross-contamination and spurious interactions occurring in samples with high coverage.

Supplementary Tables:

Supplementary Table 1: Gametophytes were present in 25 plastic slides:

This table recapitulates the gametophyte germinations and the different samples that were collected. The first column indicates the number of slides where lycopod gametophytes were observed (among a total of 216 slides, 54 per lycopod species). The second column indicates the number of samples that were collected (if several gametophytes were present in the same slide, they were pooled in the same samples); in addition, we indicated the number of spore samples that were collected, the number of root samples of herbaceous plants (that entered in the slides), and the number of root samples of lycopod sporophytes that were collected in the neighborhood of the experimental plots. The last column indicates the number of samples for which we successfully managed to amplify fungi using metabarcoding. A few gametophyte samples failed at amplifying fungi, probably because of their very small size (<0.3 mm).

Sample type	Species	Number of slides	Number of collected samples	Number of amplified samples
**Gametophytes**	*Diphasiastrum oellgaardii*	10	7	6
	Diphasiastrum tristachyum	4	3	1
	Diphasiastrum zeilleri	7	4	4
	Lycopodium clavatum	4	1	0
**Spores**	*Diphasiastrum oellgaardii*	NA	5	5
	Diphasiastrum tristachyum	NA	9	9
	Diphasiastrum zeilleri	NA	9	9
	Lycopodium clavatum	NA	5	5
**Herbaceous roots**	*Diphasiastrum oellgaardii*	NA	8	8
	Diphasiastrum tristachyum	NA	5	5
	Diphasiastrum zeilleri	NA	6	6
	Lycopodium clavatum	NA	4	4
**Sporophyte roots**	*Diphasiastrum alpinum*	NA	7	7
	Diphasiastrum oellgaardii	NA	6	6
	Diphasiastrum tristachyum	NA	7	7
	Diphasiastrum zeilleri	NA	5	5
	Lycopodium clavatum	NA	6	6

Supplementary Table 2: Number of gametophytes observed at the different extractions:

Year of extraction	Species	Number of slides containing gametophytes
**2016**	*Diphasiastrum oellgaardii*	3
	Diphasiastrum tristachyum	3
	Diphasiastrum zeilleri	2
	Lycopodium clavatum	1
**2017**	*Diphasiastrum oellgaardii*	3
	Diphasiastrum tristachyum	1
	Diphasiastrum zeilleri	3
	Lycopodium clavatum	3
**2019**	*Diphasiastrum oellgaardii*	0
	Diphasiastrum tristachyum	0
	Diphasiastrum zeilleri	1
	Lycopodium clavatum	0
**2021**	*Diphasiastrum oellgaardii*	4
	Diphasiastrum tristachyum	0
	Diphasiastrum zeilleri	1
	Lycopodium clavatum	0

Supplementary Figures:

Supplementary Figure 1: Experimental design in the Hochfeld reserve:

(a) Representation of the organization of each experimental plot:

Points indicate where the plastic slides containing lycopod spores were sowed in the soil. The different shapes represent the different dates of implantation in the soil, and the colors indicate the 4 lycopod species. Crosses represent the dates of extractions of the slides from the soil. All the remaining slides were extracted in May 2021.

(b-d) Photos of one experimental plot: at the beginning of the experiment (after the mechanical stripping of the soil; **b**) and in autumn 2018 (**d**). **(c)** present a plastic slide containing spores of *Lycopodium clavatum* before being sowed in the soil. Photo credits: P. Holveck.

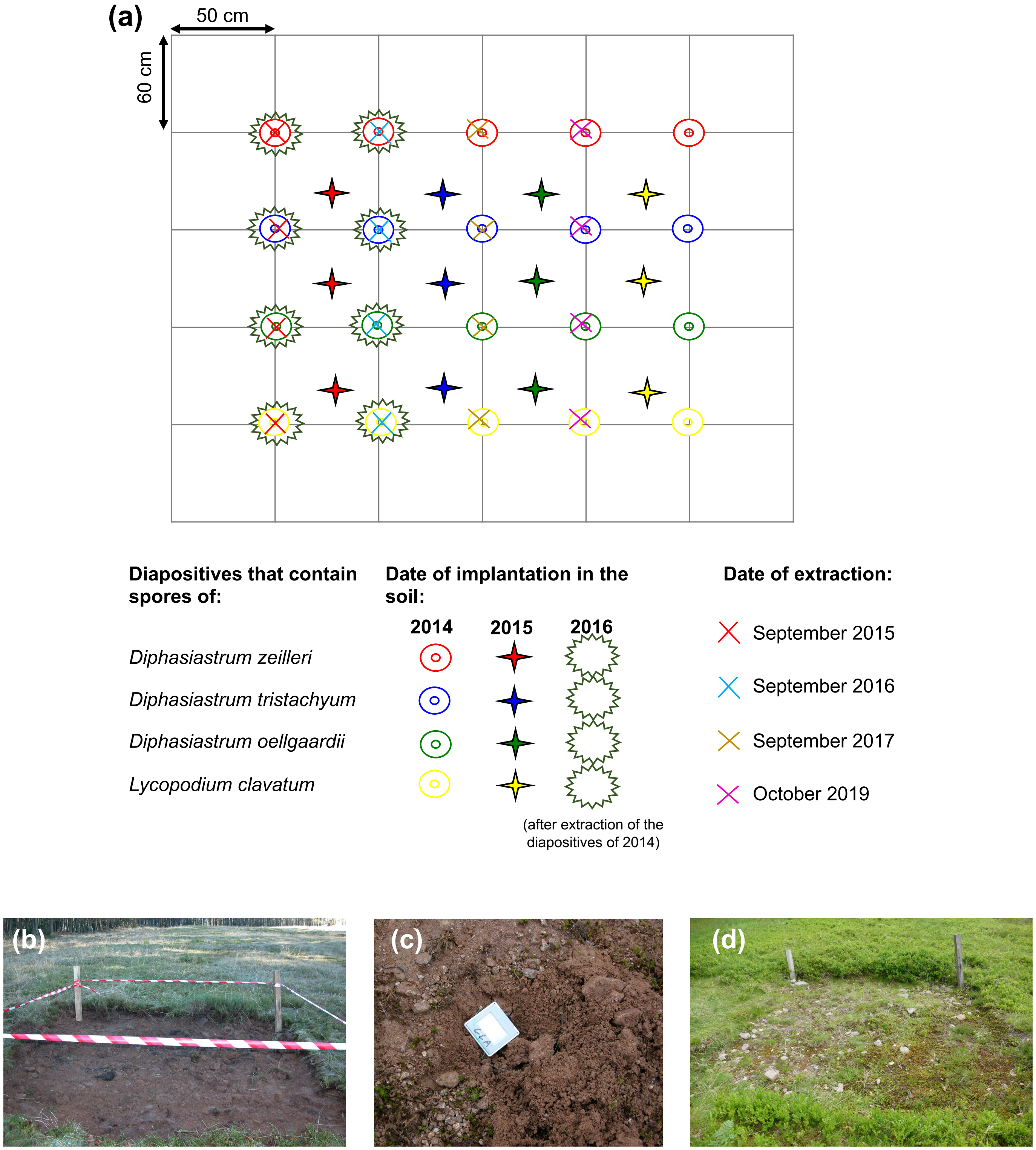
(a)
50 cm
60 cm
Diapositives that contain spores of:
Date of implantation in the soil:
Date of extraction:
2014
2015
2016
Diphasiastrum zeilleri
Diphasiastrum tristachyum
Diphasiastrum oellgaardii
Lycopodium clavatum
(after extraction of the diapositives of 2014)
September 2015
September 2016
September 2017
October 2019
(b)
(c)
(d)

Supplementary Figure 2: Mycobiome composition based on 18S rRNA metabarcoding:

Mycobiome composition in the different samples (lycopod gametophytes, lycopod spores, roots of lycopod sporophytes, or roots of herbaceous plants surrounding the gametophytes) and different lycopod species (*Lycopodium clavatum, Diphasiastrum tristachyum, Diphasiastrum oellgaardii, Diphasiastrum zeilleri,* and *Diphasiastrum alpinum*) based on 18S rRNA metabarcoding. For each sample type and each lycopod species, the number of individual samples is indicated in brackets. The bar plots represent in colors the class and the order of the main fungal lineages. Rare taxa (representing less than 0.5% of the data are represented in dark grey). Only results for the Swarm OTUs are represented (analyses based on 97% OTUs gave very similar results).

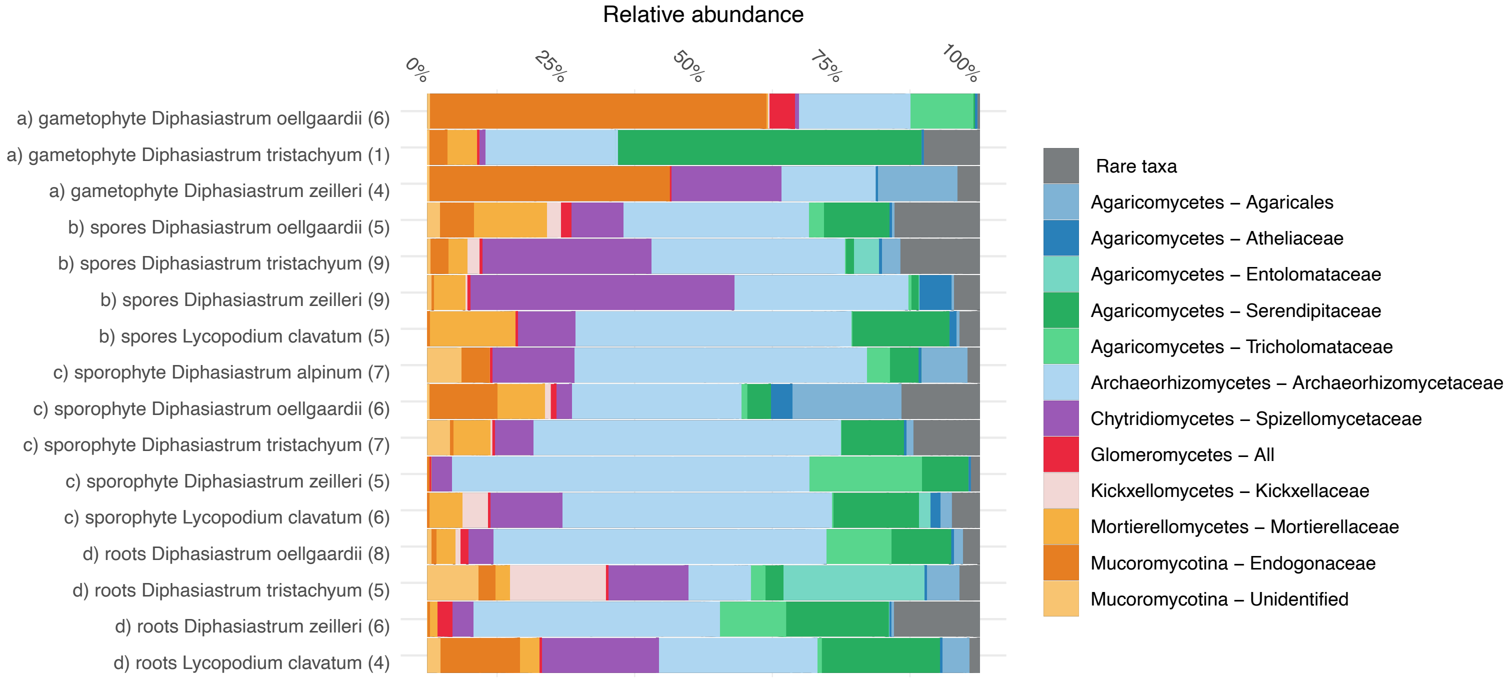

Supplementary Figure 3: Mycobiome composition based on ITS2 metabarcoding: Mycobiome composition in the different samples (lycopod gametophytes, lycopod spores, roots of lycopod sporophytes, or roots of herbaceous plants surrounding the gametophytes) and different lycopod species (*Lycopodium clavatum, Diphasiastrum tristachyum, Diphasiastrum oellgaardii, Diphasiastrum zeilleri*, and *Diphasiastrum alpinum*) based on ITS2 metabarcoding. For each sample type and each lycopod species, the number of individual samples is indicated in brackets. The bar plots represent in colors the class and the order of the main fungal lineages. Rare taxa (representing less than 0.5% of the data are represented in dark grey). Only results for the Swarm OTUs are represented (analyses based on 97% OTUs gave very similar results).

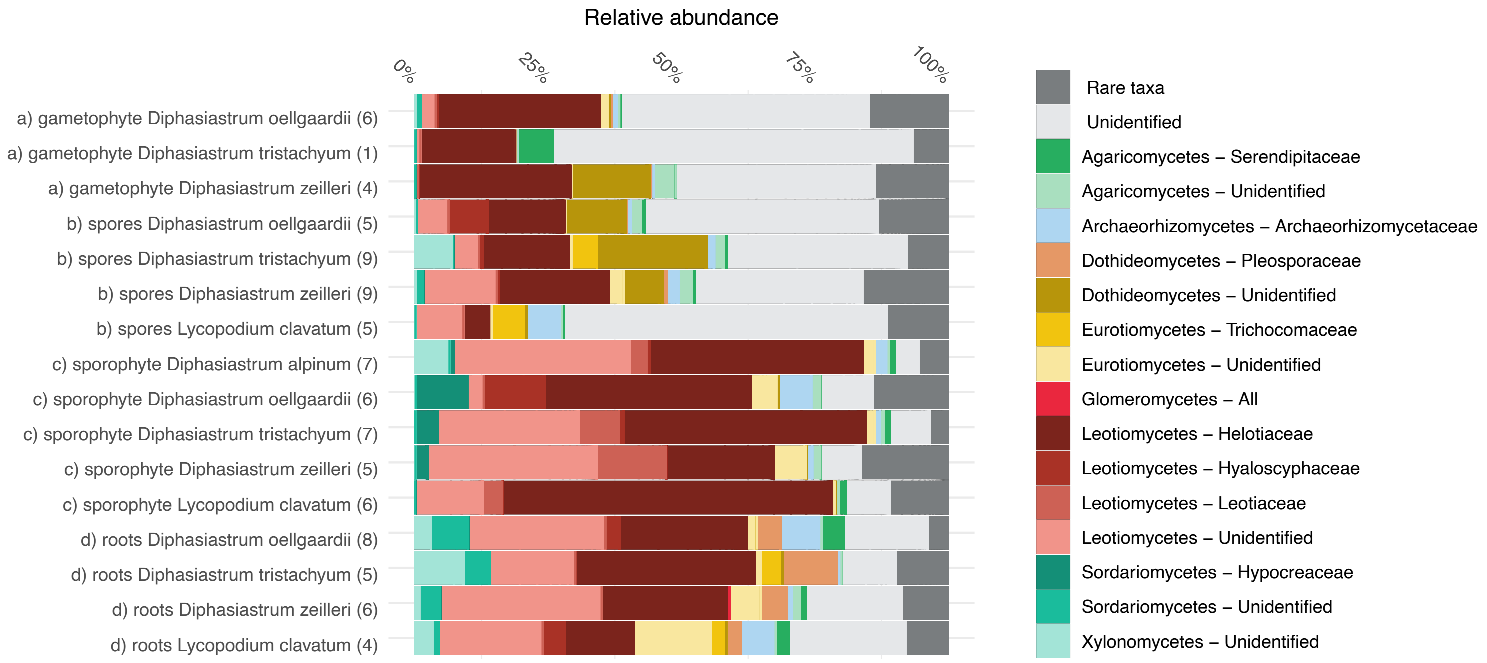

Supplementary Figure 4: Mycobiome composition significantly varies across sample types and lycopod species:

(a-b) Principal coordinate analyses (PCoA) of the mycobiomes characterized based on ITS2 (a) or 18S rRNA (b) metabarcoding. Each panel represents the projection of all the samples onto the two first axes of the PCoA performed on Bray-Curtis dissimilarities. Each sample is colored according to the sample type, while the shape indicates the lycopod species. The results of PermANOVA testing for the effect of sample type or lycopod species on mycobiome composition are indicated at the top of the panel.

(c-d) Plant-fungus interaction networks at the sample level characterized based on ITS2 (c) or 18S rRNA (d) metabarcoding. Large colored nodes represent individual samples and small grey nodes correspond to fungal OTUs. Grey links represent plant-fungus interactions and their widths are proportional to interaction abundances. The position of the nodes reflects the similarity in species interactions using the Fruchterman-Reingold layout algorithm (Fruchterman and Reingold, 1991).

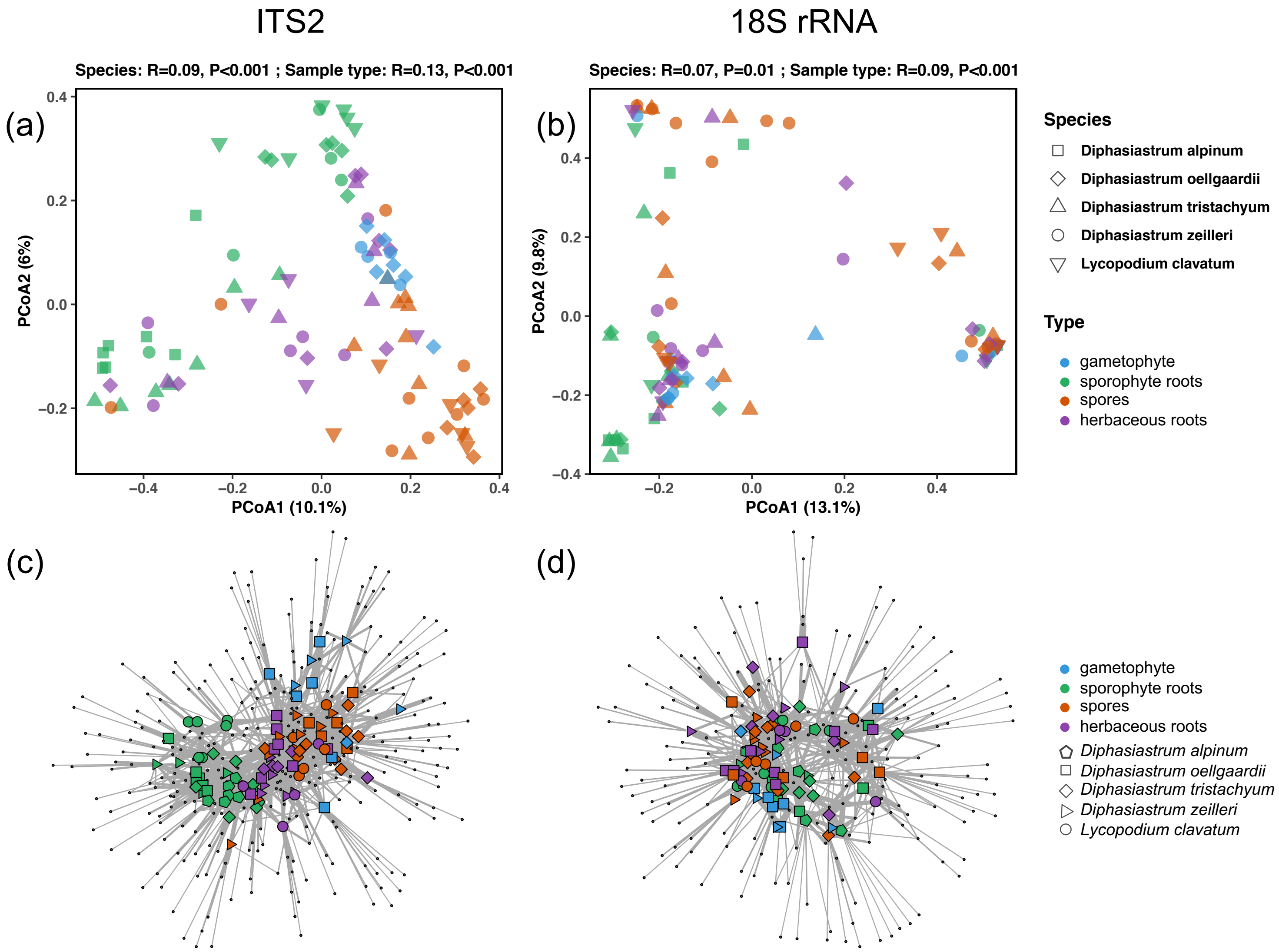

Supplementary Figure 5: Mucoromycotina OTUs are shared between lycopod gametophytes and sporophytes:

(a) Principal coordinate analyses (PCoA) of the sets of Mucoromycotina OTUs associated with each sample characterized using 18S rRNA metabarcoding. It represents the projection of all the samples onto the two first axes of the PCoA performed on Bray-Curtis dissimilarities. Each sample is colored according to the sample type, while the shape indicates the different lycopod species. The results of PermANOVA testing for the effect of sample type or lycopod species on Mucoromycotina composition are indicated at the top of the panel.

(b) Venn diagram of the Mucoromycotina OTUs between gametophytes of different lycopod species: Mucoromycotina OTUs are largely shared between gametophytes of different species.

(c) Venn diagram of the Mucoromycotina OTUs between different sample types: Mucoromycotina OTUs are largely shared between gametophytes and sporophytes, but the Mucoromycotina OTUs found in samples of spores or herbaceous roots almost never colonize gametophytes.

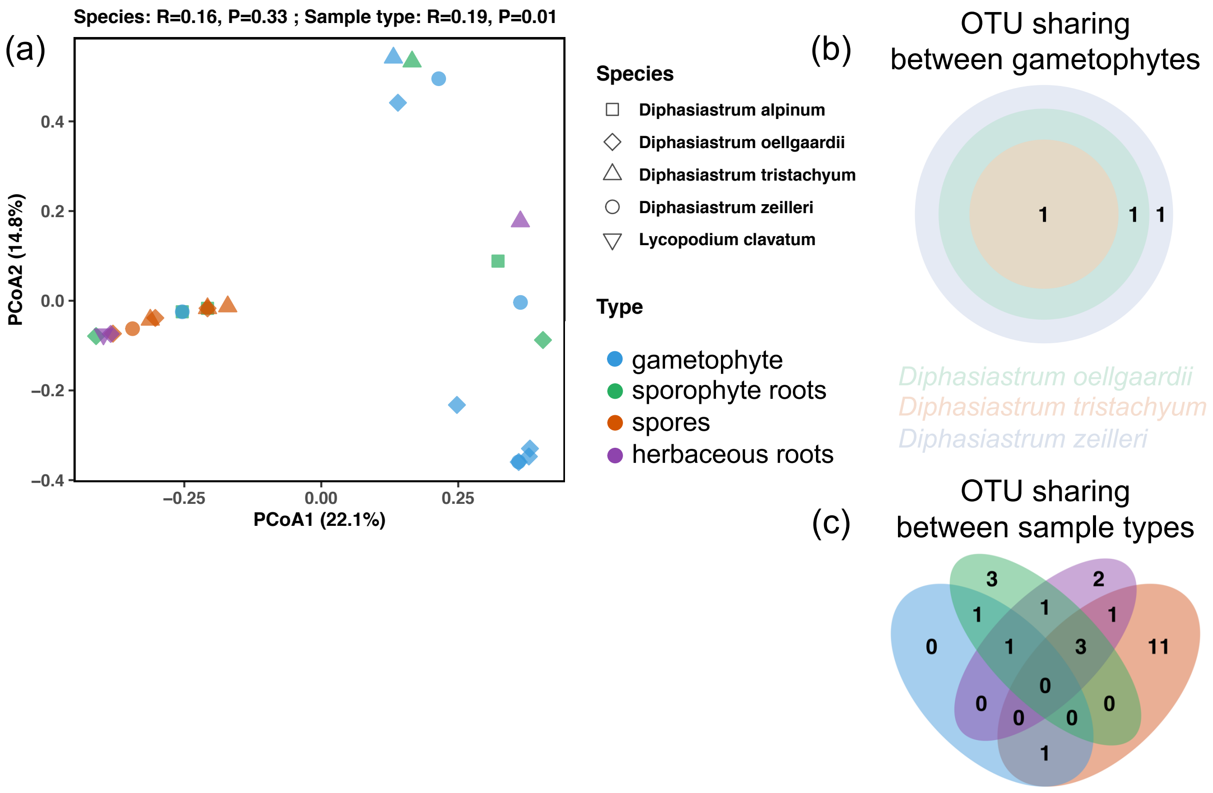

Supplementary Figure 6: Sebacinales OTUs are poorly shared between sample types and lycopod species:

(a) Principal coordinate analyses (PCoA) of the sets of Sebacinales OTUs associated with each sample characterized using ITS metabarcoding. It represents the projection of all the samples onto the two first axes of the PCoA performed on Bray-Curtis dissimilarities. Each sample is colored according to the sample type, while the shape indicates the different lycopod species. The results of PermANOVA testing for the effect of sample type or lycopod species on Sebacinales composition are indicated at the top of the panel.

(b) Venn diagram of the Sebacinales OTUs between gametophytes of different lycopod species: Sebacinales OTUs are not shared between gametophytes of different species.

(c) Venn diagram of the Sebacinales OTUs between different sample types: Many Sebacinales OTUs are not shared between samples from different types.

(d) Plant-Sebacinales interaction network at the sample-level characterized using 18S rRNA metabarcoding. Large colored nodes represent individual samples and small grey nodes correspond to fungal OTUs. Grey links represent plant-fungus interactions and their widths are proportional to interaction abundances. The position of the nodes reflects the similarity in species interactions using the Fruchterman-Reingold layout algorithm (Fruchterman and Reingold, 1991).

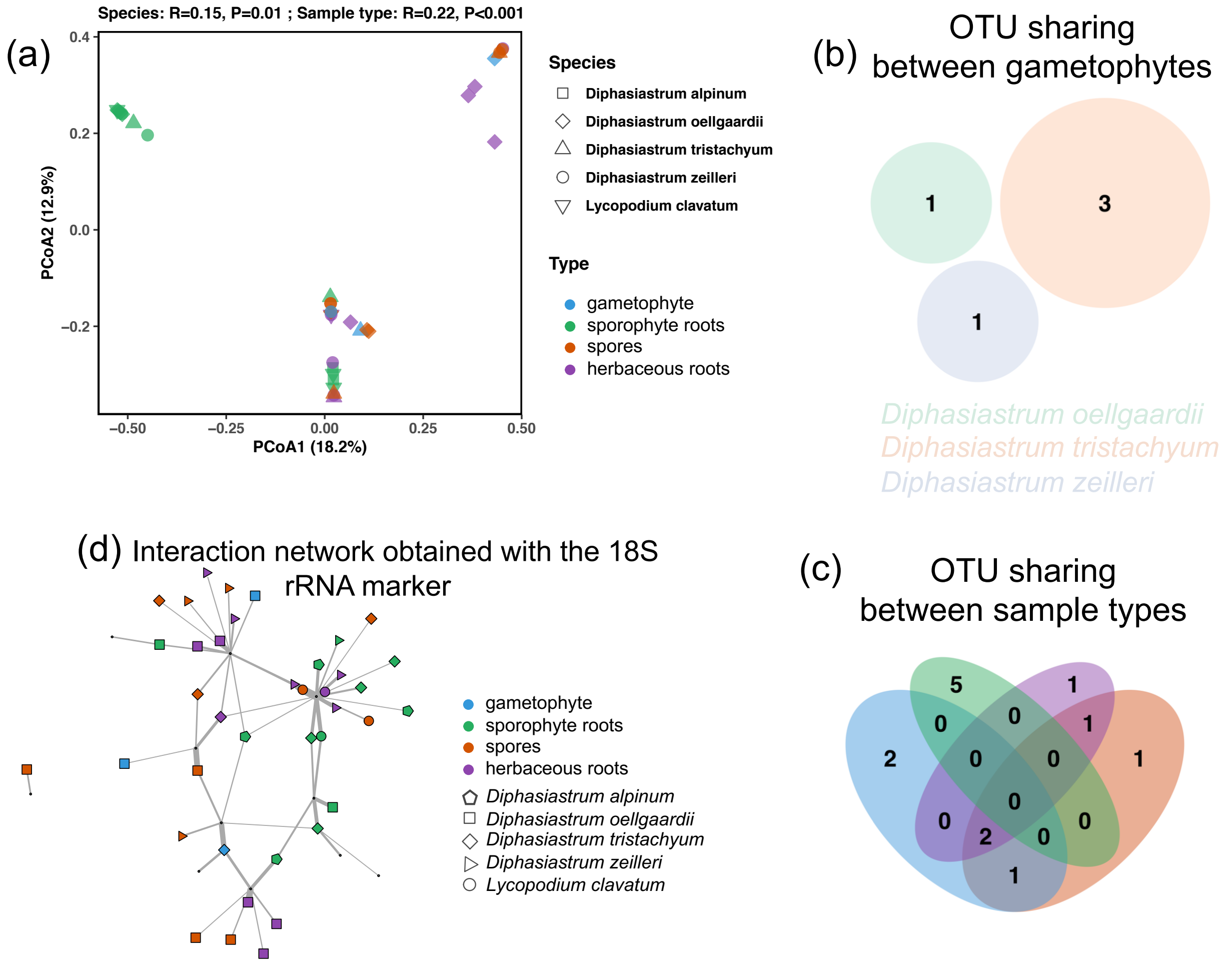
Species: R=0.15, P=0.01 ; Sample type: R=0.22, P<0.001
(a)
PCoA2 (12.9%)
PCoA1 (18.2%)
0.4
0.2
0.0
−0.2
−0.50
−0.25
0.00
0.25
0.50
Species
Diphasiastrum alpinum
Diphasiastrum oellgaardii
Diphasiastrum tristachyum
Diphasiastrum zeilleri
Lycopodium clavatum
Type
gametophyte
sporophyte roots
spores
herbaceous roots
(b)
OTU sharing
between gametophytes
1
3
1
Diphasiastrum oellgaardii
Diphasiastrum tristachyum
Diphasiastrum zeilleri
(d) Interaction network obtained with the 18S rRNA marker
gametophyte
sporophyte roots
spores
herbaceous roots
Diphasiastrum alpinum
Diphasiastrum oellgaardii
Diphasiastrum tristachyum
Diphasiastrum zeilleri
Lycopodium clavatum
(c)
OTU sharing
between sample types
5
1
0
0
1
2
0
0
1
0
0
2
0
0
1

Supplementary Figure 7: Helotiales OTUs are poorly shared between sample types and lycopod species:

(a) Principal coordinate analyses (PCoA) of the sets of Helotiales OTUs associated with each sample characterized using ITS metabarcoding. It represents the projection of all the samples onto the two first axes of the PCoA performed on Bray-Curtis dissimilarities. Each sample is colored according to the sample type, while the shape indicates the different lycopod species. The results of PermANOVA testing for the effect of sample type or lycopod species on Helotiales composition are indicated at the top of the panel.

(b) Venn diagram of the Helotiales OTUs between gametophytes of different lycopod species: Helotiales OTUs are poorly shared between gametophytes of different species.

(c) Venn diagram of the Helotiales OTUs between different sample types: Many Helotiales OTUs are not shared between samples from different types.

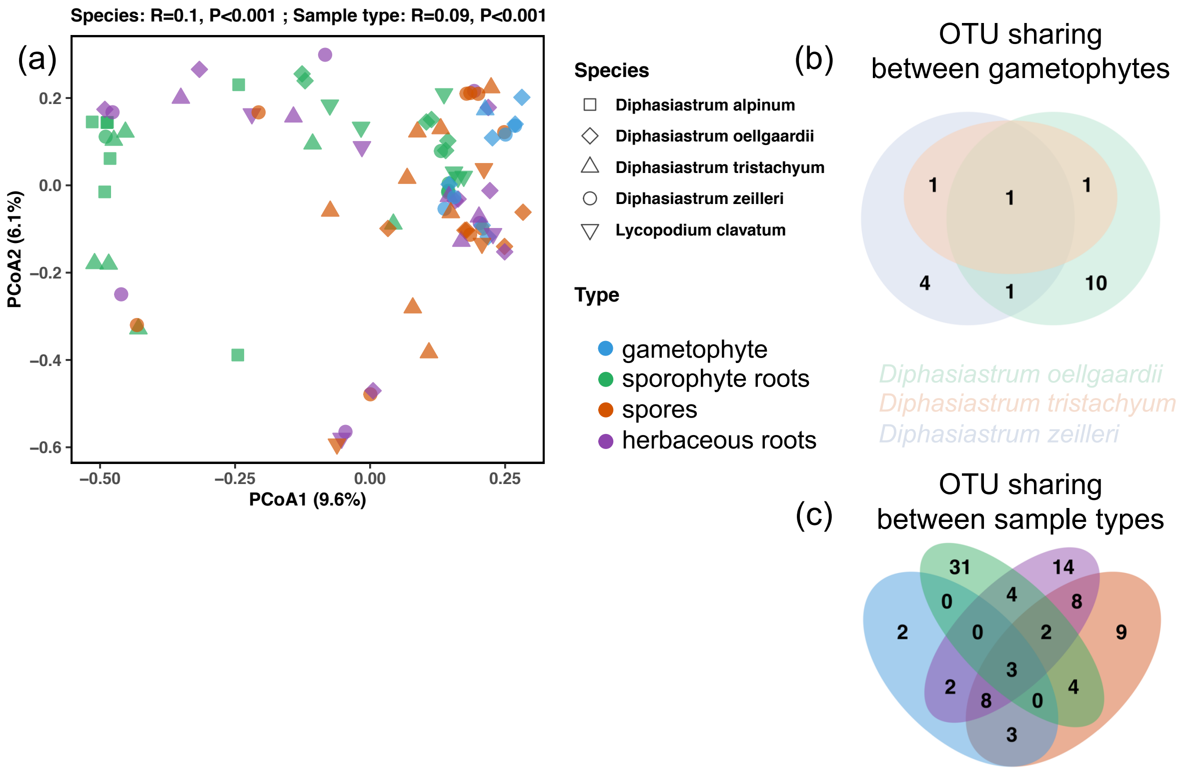

Supplementary Figure 8: One Chytridiomycota OTU is largely shared between sample types and lycopod species:

(a) Principal coordinate analyses (PCoA) of the sets of Chytridiomycota OTUs associated with each sample characterized using 18S rRNA metabarcoding. It represents the projection of all the samples onto the two first axes of the PCoA performed on Bray-Curtis dissimilarities. Each sample is colored according to the sample type, while the shape indicates the different lycopod species. The results of PermANOVA testing for the effect of sample type or lycopod species on Chytridiomycota composition are indicated at the top of the panel.

(b) Venn diagram of the Chytridiomycota OTUs between gametophytes of different lycopod species: one Chytridiomycota OTUs is shared between all lycopod species.

(c) Venn diagram of the Chytridiomycota OTUs between different sample types: Chytridiomycota OTUs are quite shared sample types and are especially abundant in spores.

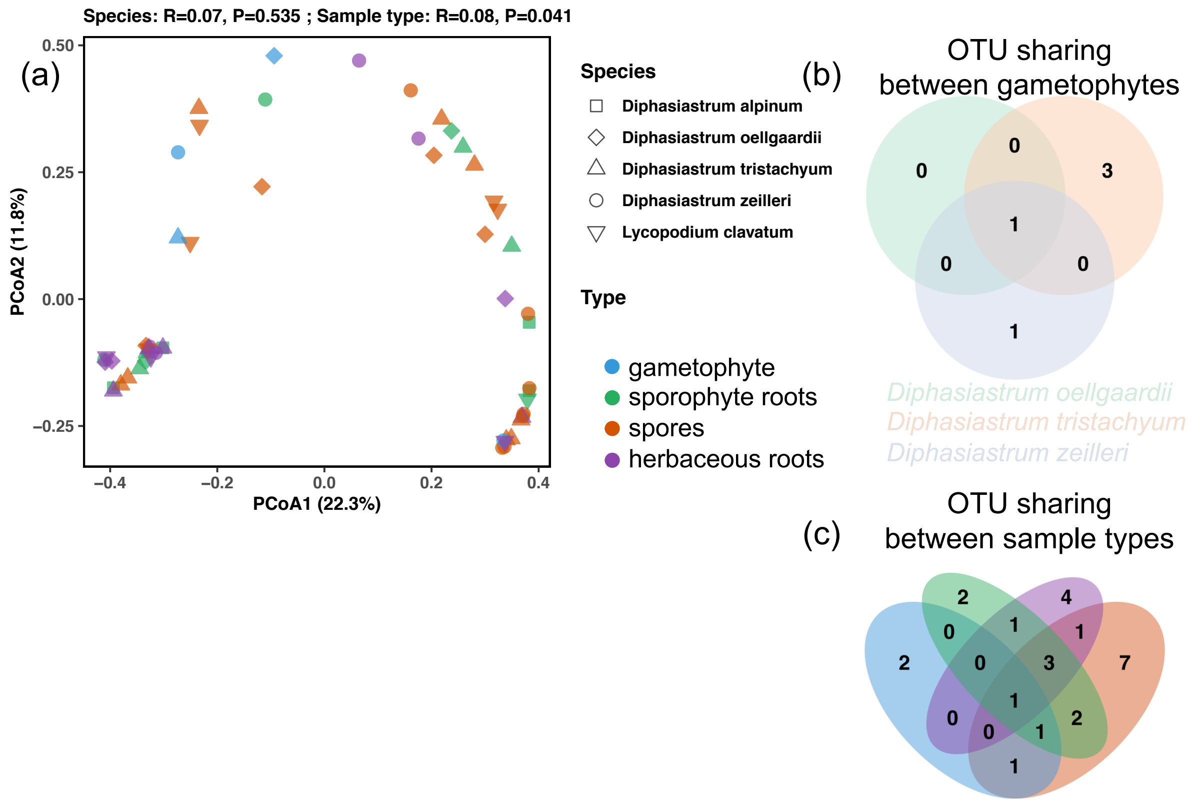

Supplementary Figure 9: When using 97% OTU clustering instead of Swarm clustering, the relative composition of the mycobiomes associated with the different sample types also revealed that Lycopodiaceae gametophytes are abundantly colonized by Endogonales (Mucoromycotina) while spores are parasitized by Chytridiomycota.

Mycobiome composition in the different samples (lycopod gametophytes, lycopod spores, roots of lycopod sporophytes, or roots of herbaceous plants surrounding the gametophytes) based on 18S rRNA or ITS metabarcoding. For each sample type, the number of individual samples is indicated in brackets. The bar plots represent in colors the class and the order of the main fungal lineages. Rare taxa (representing less than 0.5% of the data are represented in dark grey). Only results for the 97% OTUs are represented here; analyses based on Swarm OTUs gave very similar results (see Figure 3).

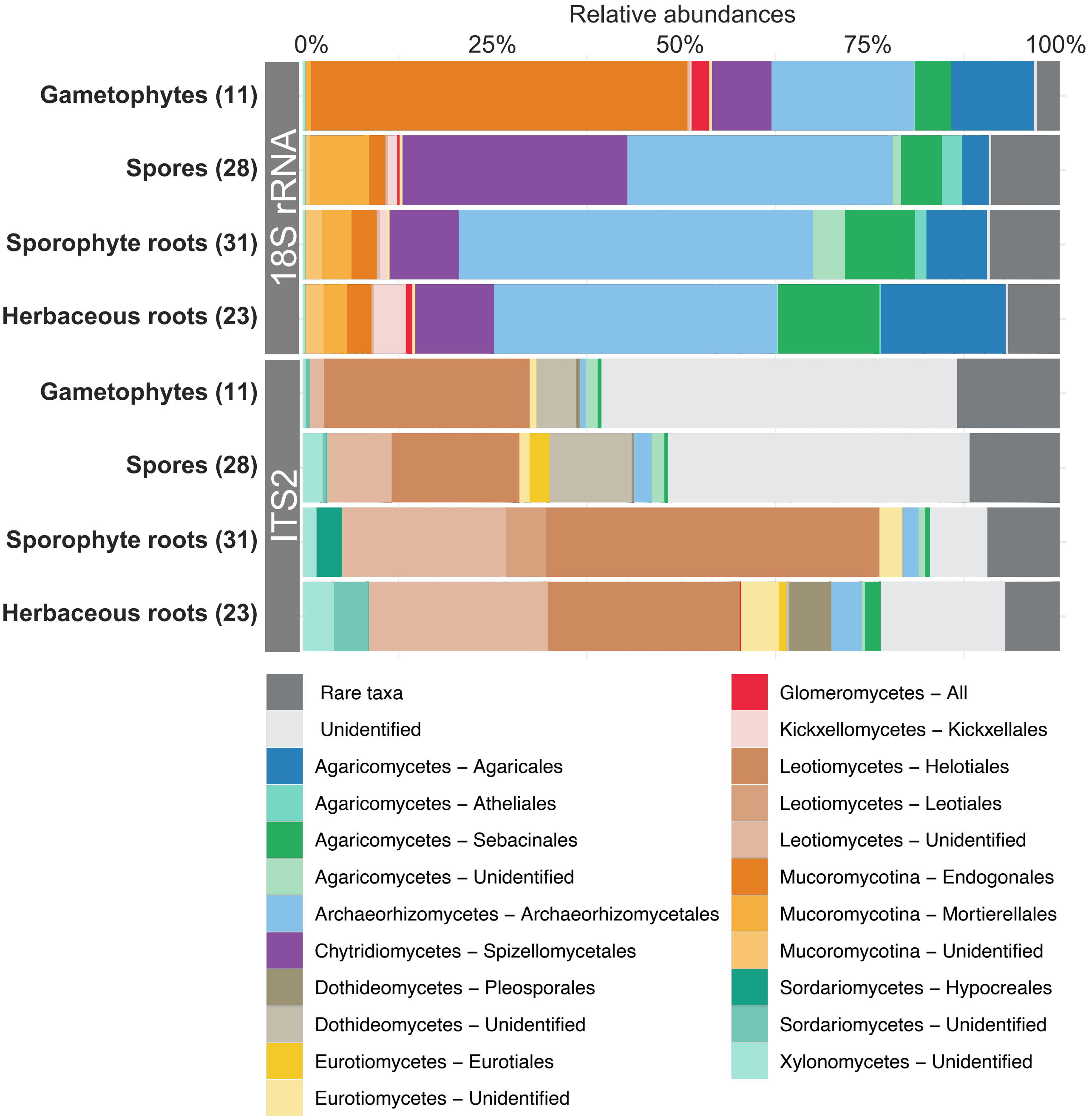

Relative abundances
0%
25%
50%
75%
100%
Gametophytes (11)
Spores (28)
Sporophyte roots (31)
Herbaceous roots (23)
18S rRNA
ITS2
Rare taxa
Unidentified
Agaricomycetes – Agaricales
Agaricomycetes – Atheliales
Agaricomycetes – Sebacinales
Agaricomycetes – Unidentified
Archaeorhizomycetes – Archaeorhizomycetales
Chytridiomycetes – Spizellomycetales
Dothideomycetes – Pleosporales
Dothideomycetes – Unidentified
Eurotiomycetes – Eurotiales
Eurotiomycetes – Unidentified
Glomeromycetes – All
Kickxellomycetes – Kickxellales
Leotiomycetes – Helotiales
Leotiomycetes – Leotiales
Leotiomycetes – Unidentified
Mucoromycotina – Endogonales
Mucoromycotina – Mortierellales
Mucoromycotina – Unidentified
Sordariomycetes – Hypocreales
Sordariomycetes – Unidentified
Xylonomycetes – Unidentified

Supplementary Figure 10: Photos of the sporophytes of the 6 lycopods species currently present in the Hochfeld reserve:

Photo credits: P. Holveck.

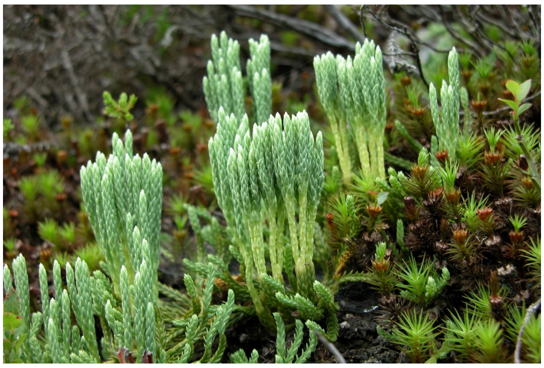

(a) ***Diphasiastrum alpinum***

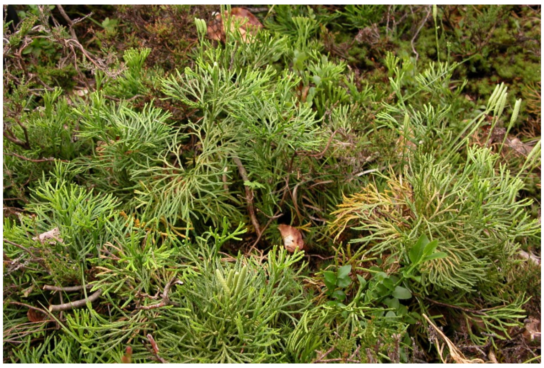

(b) ***Diphasiastrum zeilleri***

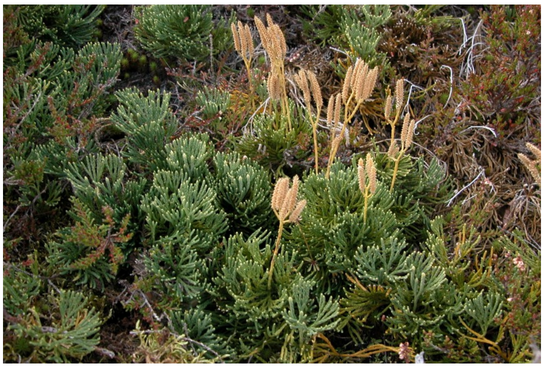

(c) ***Diphasiastrum tristachyum***

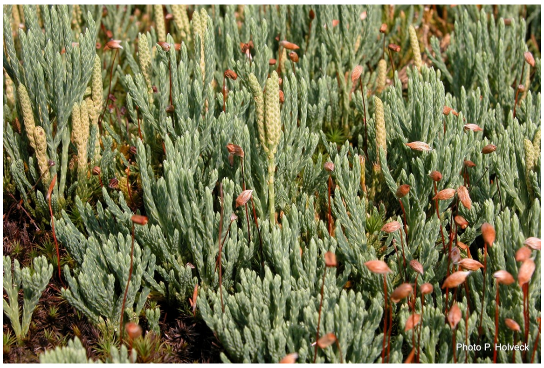

(d) ***Diphasiastrum oellgaardii***

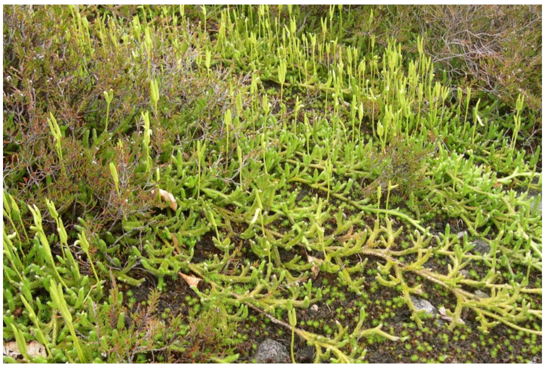

(e) ***Lycopodium clavatum***

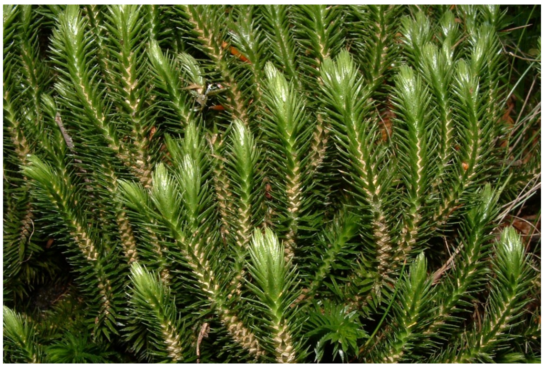

(f) ***Huperzia selago***

Supplementary references:

Berruti, A., Desirò, A., Visentin, S., Zecca, O., and Bonfante, P. (2017) ITS fungal barcoding primers versus 18S AMF-specific primers reveal similar AMF-based diversity patterns in roots and soils of three mountain vineyards. *Environ Microbiol Rep* **9**: 658–667.

Boeuf, R. (2001) La Lande à lycopodes du Hochfeld (Bas-Rhin) : quelques éléments essentiels pour la gestion d'un milieu remarquable. *Rev For Française* 252–262.

Capella-Gutierrez, S., Silla-Martinez, J.M., and Gabaldon, T. (2009) trimAl: a tool for automated alignment trimming in large-scale phylogenetic analyses. *Bioinformatics* **25**: 1972–1973.

Davis, N.M., Proctor, D.M., Holmes, S.P., Relman, D.A., and Callahan, B.J. (2018) Simple statistical identification and removal of contaminant sequences in marker-gene and metagenomics data. *Microbiome* **6**: 226.

Durr-Ecolor, T. (2021) Lande à Lycopodes du Hochfeld au Hohwald (420030413).

Fruchterman, T.M.J. and Reingold, E.M. (1991) Graph drawing by force-directed placement. *Softw Pract Exp* **21**: 1129–1164.

Hoysted, G.A., Bidartondo, M.I., Duckett, J.G., Pressel, S., and Field, K.J. (2021) Phenology and function in lycopod–Mucoromycotina symbiosis. *New Phytol* **229**: 2389–2394.

Kalyaanamoorthy, S., Minh, B.Q., Wong, T.K.F., von Haeseler, A., and Jermiin, L.S. (2017) ModelFinder: fast model selection for accurate phylogenetic estimates. *Nat Methods* **14**: 587–589.

Katoh, K. and Standley, D.M. (2013) MAFFT Multiple sequence alignment software version 7: Improvements in performance and usability. *Mol Biol Evol* **30**: 772–780.

Mahé, F., Rognes, T., Quince, C., de Vargas, C., and Dunthorn, M. (2015) Swarmv2: Highly-scalable and high-resolution amplicon clustering. *PeerJ* **2015**: 1–12.

Martin, M. (2011) Cutadapt removes adapter sequences from high-throughput sequencing reads. *EMBnet.journal* **17**: 10.

Nguyen, L.T., Schmidt, H.A., Von Haeseler, A., and Minh, B.Q. (2015) IQ-TREE: A fast and effective stochastic algorithm for estimating maximum-likelihood phylogenies. *Mol Biol Evol* **32**: 268–274.

Nilsson, R.H., Larsson, K.H., Taylor, A.F.S., Bengtsson-Palme, J., Jeppesen, T.S., Schigel, D., et al. (2019) The UNITE database for molecular identification of fungi: Handling dark taxa and parallel taxonomic classifications. *Nucleic Acids Res* **47**: D259–D264.

Oksanen, J., Kindt, R., Pierre, L., O'Hara, B., Simpson, G.L., Solymos, P., et al. (2016) vegan: Community Ecology Package, R package version 2.4-0. *R Packag version 22-1*.

Perez-Lamarque, B., Krehenwinkel, H., Gillespie, R.G., and Morlon, H. (2022) Limited evidence for microbial transmission in the phylosymbiosis between Hawaiian spiders and their microbiota. *mSystems* **7**: e01104-21.

Perez-Lamarque, B., Petrolli, R., Strullu-Derrien, C., Strasberg, D., Morlon, H., Selosse, M.-A., and Martos, F. (2022) Structure and specialization of mycorrhizal networks in phylogenetically diverse tropical communities. *Environ Microbiome* **17**: 38.

Petrolli, R., Augusto Vieira, C., Jakalski, M., Bocayuva, M.F., Vallé, C., Cruz, E.D.S., et al. (2021) A fine-scale spatial analysis of fungal communities on tropical tree bark unveils the epiphytic rhizosphere in orchids. *New Phytol* **231**: 2002–2014.

Quast, C., Pruesse, E., Yilmaz, P., Gerken, J., Schweer, T., Yarza, P., et al. (2013) The SILVA ribosomal RNA gene database project: Improved data processing and web-based tools. *Nucleic Acids Res* **41**: D590–D596.

Rimington, W.R., Pressel, S., Duckett, J.G., and Bidartondo, M.I. (2015) Fungal associations of basal vascular plants: reopening a closed book? *New Phytol* **205**: 1394–1398.

Rimington, W.R., Pressel, S., Duckett, J.G., Field, K.J., Read, D.J., and Bidartondo, M.I. (2018) Ancient plants with ancient fungi: liverworts associate with early-diverging arbuscular mycorrhizal fungi. *Proc R Soc B Biol Sci* **285**: 20181600.

Rognes, T., Flouri, T., Nichols, B., Quince, C., and Mahé, F. (2016) VSEARCH: A versatile open source tool for metagenomics. *PeerJ* **2016**: e2584.

Taberlet, P., Bonin, A., Zinger, L., and Coissac, E. (2018) DNA amplification and multiplexing. In *Environmental DNA*. pp. 41–57.

Toju, H., Guimarães, P.R., Olesen, J.M., and Thompson, J.N. (2014) Assembly of complex plant-fungus networks. *Nat Commun* **5**: 1–7.

Turenne, C.Y., Sanche, S.E., Hoban, D.J., Karlowsky, J.A., and Kabani, A.M. (1999) Rapid identification of fungi by using the ITS2 genetic region and an automated fluorescent capillary electrophoresis system. *J Clin Microbiol* **37**: 1846–1851.

White, T.J.T., Bruns, T., Lee, S., and Taylor, J. (1990) Amplification and direct sequencing of fungal ribosomal RNA genes for phylogenetics. In *PCR Protocols*. Elsevier, pp. 315–322.

Zinger, L., Bonin, A., Alsos, I.G., Bálint, M., Bik, H., Boyer, F., et al. (2019) DNA metabarcoding—Need for robust experimental designs to draw sound ecological conclusions. *Mol Ecol* **28**: 1857–1862.
